# A Novel Siglec-4 Derived Spacer Improves the Functionality of CAR T Cells Against Membrane-Proximal Epitopes

**DOI:** 10.3389/fimmu.2020.01704

**Published:** 2020-08-07

**Authors:** Daniel Schäfer, Janina Henze, Rita Pfeifer, Anna Schleicher, Janina Brauner, Nadine Mockel-Tenbrinck, Carola Barth, Daniela Gudert, Wa'el Al Rawashdeh, Ian C. D. Johnston, Olaf Hardt

**Affiliations:** ^1^Translational Molecular Imaging, Institute for Diagnostic and Interventional Radiology & Clinic for Hematology and Medical Oncology, University Medical Center Göttingen, Göttingen, Germany; ^2^R&D Reagents, Miltenyi Biotec B.V. & Co. KG, Bergisch Gladbach, Germany; ^3^Faculty of Chemistry and Biosciences, Karlsruher Institute of Technology, Karlsruhe, Germany

**Keywords:** chimeric antigen receptor, hinge, spacer, Siglec, CH2-CH3, IgG, CAR design

## Abstract

A domain that is often neglected in the assessment of chimeric antigen receptor (CAR) functionality is the extracellular spacer module. However, several studies have elucidated that membrane proximal epitopes are best targeted through CARs comprising long spacers, while short spacer CARs exhibit highest activity on distal epitopes. This finding can be explained by the requirement to have an optimal distance between the effector T cell and target cell. Commonly used long spacer domains are the CH2-CH3 domains of IgG molecules. However, CARs containing these spacers generally show inferior *in vivo* efficacy in mouse models compared to their observed *in vitro* activity, which is linked to unspecific Fcγ-Receptor binding and can be abolished by mutating the respective regions. Here, we first assessed a CAR therapy targeting membrane proximal CD20 using such a modified long IgG1 spacer. However, despite these mutations, this construct failed to unfold its observed *in vitro* cytotoxic potential in an *in vivo* model, while a shorter but less structured CD8α spacer CAR showed complete tumor clearance. Given the shortage of well-described long spacer domains with a favorable functionality profile, we designed a novel class of CAR spacers with similar attributes to IgG spacers but without unspecific off-target binding, derived from the Sialic acid-binding immunoglobulin-type lectins (Siglecs). Of five constructs tested, a Siglec-4 derived spacer showed highest cytotoxic potential and similar performance to a CD8α spacer in a CD20 specific CAR setting. In a pancreatic ductal adenocarcinoma model, a Siglec-4 spacer CAR targeting a membrane proximal (TSPAN8) epitope was efficiently engaged *in vitro*, while a membrane distal (CD66c) epitope did not activate the T cell. Transfer of the TSPAN8 specific Siglec-4 spacer CAR to an *in vivo* setting maintained the excellent tumor killing characteristics being indistinguishable from a TSPAN8 CD8α spacer CAR while outperforming an IgG4 long spacer CAR and, at the same time, showing an advantageous central memory CAR T cell phenotype with lower release of inflammatory cytokines. In summary, we developed a novel spacer that combines cytotoxic potential with an advantageous T cell and cytokine release phenotype, which make this an interesting candidate for future clinical applications.

## Introduction

The unprecedented therapeutic efficacy of CAR T cells in previously refractory blood cancers is considered to be one of the major breakthroughs in cancer immunotherapy, culminating in the recent market approvals by the Food and Drug Administration (FDA) and the European Medicines Agency (EMA) for two CAR T cell products (1–7). While CAR therapies have now achieved public recognition, their development and the quest for optimal CAR design has been a multistep process stretching over several decades. Ever since their initial description in 1989 by Eshhar et al. (8), the receptors have evolved from a two-chimeric-TCR chain architecture to a one-protein design. This design commonly incorporates a single-chain variable fragment (scFv) of a given antibody as the antigen binding moiety, an extracellular spacer and a transmembrane region as structural features, as well as signal transduction units for T cell activation. Originally, the spacer domain was introduced into the CAR framework as an inert building block to allow the antigen binding moiety to extend beyond the T cell's glycocalyx and improve antigen accessibility (9). Following this assumption, a plethora of spacer regions were designed simultaneously ranging from the immunoglobulin (Ig) domains of the crystallizable fragments (Fc) of antibodies to extracellular domains of CD8α, CD28, the TCRβ chain or NKG2D (10–16) and were applied without comparative analyses. However, already very early on, Patel and colleagues provided the scientific proof that the spacer region can be of paramount importance for the receptor function and affects its expression, surface stability through the turnover rate, and signal transduction (17). More recent accumulating research has further been showing that in addition to the nature of the spacer, effective antigen recognition depends on the functional interplay between the spatial localization of the target epitope and the CAR spacer length (18–20). For instance, membrane-distal epitopes were shown to most efficiently trigger CARs with short spacers, while membrane-proximal epitopes required receptors with extended spacer domains to elicit accurate effector function, in this way emphasizing the biological requirement of optimal T cell-target cell distance (18–22). Thus, the design of CARs against novel antigens needs to consider both the epitope position within the target antigen as well as the nature and length of the spacer region and customize these variables accordingly.

The use of Ig-derived spacers is particularly attractive as it provides the opportunity to modulate the spacer length into long (CH2-CH3 domain), medium (CH3) and short (hinge only) structures, while retaining the nature of the parent protein. However, Ig-derived spacers have faced various complications during their development. In particular, off-target activation, CAR T cell sequestration in the lung, tonic signaling and activation-induced cell death (AICD) have been described leading to only a limited T cell persistence (23–26). Although these effects could be abrogated by mutating the amino acid sequence essential for FcR binding (23, 25, 27), it needs to be taken into consideration that these experiments were conducted in immunosuppressed NSG mice and whether FcR binding can be entirely eliminated in humans remains unclear. Of note, several clinical studies that used IgG-derived spacers described only limited anti-tumor efficacy and low CAR T cell persistence (28–31) while others are showing some promising clinical responses (32–34). Interestingly, the first commercially available CAR T cell-based therapies use CD28 (Yescarta) and CD8 (Kymriah) derived spacer domains.

Taking into account the shortage of well-described long spacer domains with a favorable functionality profile, we endeavored to develop a novel long spacer for membrane-proximal epitopes, which naturally lacks an FcR binding domain. Based on the postulated spatial requirements between CARs and their target antigens, we anticipated finding a CAR spacer construct whose functionality against membrane-proximal epitopes extends beyond that of a CD8α spacer CAR. Hence, we generated novel CAR spacers and analyzed their efficacy side-by-side to the cognate CD8α spacer counterpart – a comparison that has not been extensively undertaken thus far. The design of the novel spacers was based on the sialic acid binding Ig-like lectin (Siglec) receptor family, whose members are broadly expressed on various immune cells (35, 36). Structurally, each receptor member is composed of an N-terminal Ig-like V-set domain which is involved in sialic acid binding and a defined number of Ig-like C2-set domains that serve as a structural spacer and extend the binding moiety away from the plasma membrane. The selection of the Siglec family was inspired by the hypothesis that the incorporation of naturally occurring spacer domains into the CAR architecture will preserve the biological requirements of a spacer region and minimize unspecific interactions with other cells.

In this study, we confirm this strategy of using naturally occurring spacer domains by first demonstrating, that in a CD20^+^ lymphoma model a long IgG1 spacer CAR is as functional as the CD8α spacer *in vitro*, but fails to translate its effectiveness *in vivo*, despite containing the earlier reported mutations to abrogate FcR binding (23). Subsequently, we evaluate novel spacers derived from the Siglec family of proteins and identify a long alternative spacer derived from Siglec-4 that performs with equal efficiency to the CD8α spacer *in vitro*. Finally, we demonstrate in a solid tumor model that the novel Siglec-4 spacer CAR does not exceed, but rather matches the CD8α spacer CAR cytotoxic activity *in vivo* on membrane-proximal targets, while maintaining a favorable cell phenotype profile and cytokine release pattern.

## Materials and Methods

### CAR Gene Construction

Commercial gene synthesis in combination with an optimization algorithm for codon usage in humans (ATUM) was used to construct the *CAR* genes of interest. The CD20-specific scFv was derived from the murine monoclonal antibody Leu16 as originally described by Jensen and colleagues (37), while the CD66c- and TSPAN8-targeting scFv sequences were derived from the antibody clones REA414 (CD66c) and REA443 (TSPAN8) (Miltenyi Biotec). All antigen binding domains contained a (G_4_S)_3_-linker between the V_L_ and the V_H_ regions. To facilitate receptor trafficking to the plasma membrane, a human CD8α leader signaling peptide was added N-terminally to the respective scFv sequence. The spacer region downstream of the scFv encompassed either the domain for IgG1 hinge-CH2CH3 (234 amino acids), IgG4 hinge-CH2CH3 (228 amino acids), or CD8α hinge (45 amino acids). To abrogate potential interactions of the Fc spacer CARs with FcR-expressing cells, the PELLGG and ISR motives in the IgG1 CH2 domain were replaced by the corresponding IgG2 amino acids (23). In the case of the IgG4 CH2 domain, the APEFLG sequence was replaced by APPVA from IgG2 and an N279Q mutation was introduced to remove glycosylation at this site (25). Spacers derived from the Siglec family were designed based on the protein sequences extracted from UniProt and the plasma membrane-proximal domains were incorporated into the CAR architecture. Thus, the Siglec-3 spacer comprised the amino acids 145–259 of the parent protein with a C169S mutation to abrogate unspecific disulfide-bond formation. The Siglec-4 spacer contained the amino acids 238–519, the Siglec-7.1 spacer the amino acids 150–353, the Siglec-7.2 spacer the amino acids 234–353, and the Siglec-8 spacer the amino acids 241–363 of the respective parent protein. All spacers were linked to the transmembrane domain of human CD8α, the intracellular domain of 4-1BB, and the CD3ζ signaling domain as derived from UniProt. The *CAR* genes were fused to a Furin-P2A sequence to include co-expression of the truncated low affinity nerve growth factor receptor (ΔLNGFR). Transgene expression was promoted by the PGK promoter located upstream of the *CAR* gene.

### Lentiviral Vector Production

Second generation self-inactivating VSV-G-pseudotyped lentiviral vectors were produced by transient transfection of adherent HEK293T cells. One day before transfection, 1.6 × 10^7^ HEK293T cells were seeded per T175 flask to reach a confluency of 70–90% on the following day. Each T175 flask was then transfected with a total of 35 μg plasmid DNA composed of pMDG2 (encoding VSV-G), pCMVdR8.74 (encoding gag/pol), and the respective transgene-encoding transfer vector using MACSfectin reagent (Miltenyi Biotec). All transfection reactions were performed with a DNA: MACSfectin ratio of 1:2. Following overnight incubation, sodium butyrate was supplied at a final concentration of 10 mM and at 48 h after transfection the medium was collected, cleared by centrifugation at 300× g and 4°C for 5 min and filtered through 0.45 μm-pore-size PVDF filters. Concentration of the viral stock was performed by centrifugation at 4°C and 4,000 × g for 24 h. Pellets containing lentiviral vector were air-dried and resuspended at a 100-fold concentration with 4°C cold PBS. Lentiviral vector aliquots were stored at −80°C.

### Generation of CAR T Cells

#### Automated CAR T Cell Generation

The CliniMACS Prodigy® TCT (T cell transduction) application was used for the automated manufacturing of large amounts of gene-modified T cells. Technical features and experimental procedures have previously been described in detail (38, 39). In brief, T cells were obtained from non-mobilized leukapheresis from healthy anonymous donors (University Hospital Cologne or the German Red Cross Ulm) and were typically processed 24–48 h after collection. Transduced and enriched CAR T cells were finally formulated and harvested in Composol® solution (Fresenius Kabi), supplemented with 2.5% human serum albumin (Grifols). For quality assurance, the transduction efficiency and T cell phenotype was determined using a MACSQuant Analyzer 10 (Miltenyi Biotec) after the TCT process. Transduction efficiency were determined by flow cytometry on days 5 and 12 of the TCT process using a flow cytometer.

#### Manual CAR T Cell Generation

Buffy coats from healthy anonymous donors were obtained from the German Red Cross Dortmund. Peripheral blood mononuclear cells (PBMCs) were then isolated from buffy coats by density gradient centrifugation. T cells were purified from PBMCs applying the Pan T Cell Isolation Kit, human (Miltenyi Biotec) and activated in TexMACS™ Medium (Miltenyi Biotec) supplemented with T Cell TransAct™, human (Miltenyi Biotec) and 100 IU/ml of recombinant Human IL-2 IS, research grade (Miltenyi Biotec). T cells were transduced 24 h after activation using VSV-G pseudotyped lentiviral particles. 3 days post activation, T Cell TransAct™, human and excess viral vector were removed and T cells were cultured in TexMACS™ Medium only supplemented with IL-2. T cells were expanded for 12 days and used directly for *in vitro* assays or frozen in TexMACS™ Medium containing 10% DMSO for later *in vivo* use. Frozen T cells that were used for *in vivo* testing were thawed 24 h before injection and cultivated at 37°C in TexMACS™ Medium without further supplements.

#### Target Cell Lines

HEK293T, JeKo-1, Raji and AsPC1 cells were obtained from ATCC and cultured as recommended. Raji cells were transduced with with a ffLuc cassette for *in vivo* detection and AsPC1 cells were transduced with with a eGFP/ffLuc cassette for *in vitro* and *in vivo* detection. To validate authenticity of the cell lines used, we used the Human STR Profiling Cell Authentication Service (ATCC).

#### Flow Cytometry

Antibodies specific for anti-human CD62L, CD45RO, CD95, CD271 (LNGFR), CD107a, TNF-α, CD223 (LAG3), CD279 (PD1), CD366 (TIM3), CD137 (4-1BB), CD4, CD8, CD3 were monoclonal recombinant antibodies (Miltenyi Biotec). For anti-CD20 CAR detection the CD20 CAR Detection Reagent (Miltenyi Biotec) was used. Staining of Miltenyi Biotec antibodies was performed according to the supplier's instructions. For direct CAR detection of CD66c and TSPAN8 specific CARs a sequential staining was used. First, samples were incubated with polyclonal Fab specific anti-mouse IgG antibodies produced in goat (Merck) at concentrations of 10 μg/ml for 30 min at 4°C. Samples were washed and then incubated with polyclonal anti-goat IgG antibodies produced in chicken (Thermo Fisher) at concentrations of 10 μg/ml for 30 min at 4°C. Stained samples were measured on a MACSQuant® Analyzer 8 or MACSQuant Analyzer 10 (Miltenyi Biotec) and analyzed using the MACSQuantify™ Software.

### *In vitro* Functional Assays

#### With JeKo-1 Target Cells

1 × 10^5^ JeKo-1 and 1 × 10^5^ CAR T cells were co-cultured in TexMACS™ Medium (Miltenyi Biotec) for 24 h in 96-well round bottom plates. Supernatants were collected at the endpoint and used to detect the cytokines released by anti-CD20 CAR T cells using the MACSPlex Cytokine 12 Kit (Miltenyi Biotec) with the four selected human cytokines IFN-γ, IL-2, TNF-α and GM-CSF, according to the manufacturer's instructions. The cytolytic activity of the engineered T cells was evaluated by using 1 × 10^4^ CD20^+^ JeKo-1 cells labeled with 1 μM CellTrace^TM^ Violet (Life Technologies), as target cells. Effector and target cells were co-cultured for 24 h at the indicated ratios (E:T) in 96-well round bottom plates. Detection of the specific lysis was performed by quantitation of Violet dye labeled target cells using a MACSQuant Analyzer 8 (Miltenyi Biotec). Mock-transduced T cells were used as control at the same effector-to-target ratios.

#### With Raji Cells

2 × 10^5^ CAR T cells were incubated with 2 × 10^5^ CD20^+^ Raji cells in 200 μl TexMACS™ Medium at 37°C. In addition, the medium was supplemented with 20 μl of a CD107a specific antibody. After 1 h of incubation the protein transport inhibitors Monensin and Brefeldin A (BD Biosiences) were added as recommended for 4 h. After this incubation period, cells were washed and first surface stained with LNGFR specific antibodies to label transduced T cells and subsequently intracellularly stained for TNF-α using the Inside Stain Kit and a TNF-α specific antibody (all Miltenyi Biotec). Cells were then measured by flow cytometry. For TIM3, LAG3 and PD1 detection 1 × 10^5^ CAR T cells were inoculated with 2 × 10^5^ CD20^+^ Raji cells in 200 μl TexMACS™ Medium at 37°C for 24 h. Subsequently T cells were stained and analyzed by flow cytometry.

For functionality assays in the presence of NSG macrophages, 2 × 10^5^ CAR T cells were incubated in a 1:1:1 ratio with Raji target cells and macrophages derived from a peritoneal lavage. The assay was performed in the presence or absence of murine FcR-blocking reagent. After 24 h of incubation, detection of the specific lysis was performed by quantitation of Violet dye labeled target cells via flow cytometry using a MACSQuant Analyzer 8 (Miltenyi Biotec).

#### With AsPC1 Cells

GFP^+^/Luc^+^ AsPC1 target cells were inoculated in 96-well plates at 2.5 × 10^4^ cells per well in TexMACS™ Medium. CAR T cells or untransduced Mock T cells were added with at an E:T ratio of 2:1. The amount of T cells in the Mock control was adjusted to the number of total T cells in the CAR group with the highest total cell count. Cytotoxicity was measurement as the decrease of green surface area as assessed by the IncuCyte® S3 Live-Cell Analysis System (Sartorius). Measured values were normalized to the start of the experiment. After 24 h a supernatant sample was taken for cytokine measurements using the MACSPlex Cytokine 12 Kit. At the end of the experiment expression of LAG3, PD1, and 4-1BB were measured using a MACSQuant Analyzer 8 (Miltenyi Biotec). Specific endpoint killing was calculated from the green surface area values with the following formula:

$$\begin{array}{r} specific\;killing\;\left[\%\right]=\;100-\left(100{\;}^{*}\;\frac{green\;area\;Mock}{green\;area\;CAR}\;\right). \end{array}$$

#### *In vivo* Assays

Experiments involving animal handling were approved by the Governmental Review Committee on Animal Care in NRW, Germany and performed according to guidelines and regulations (Landesamt für Natur, Umwelt and Verbraucherschutz NRW, Approval number 84-02.04.2015.A168 and Approval number 84-02.04.2017.A021).

Raji lymphoma were established by tail vein injection of 5 × 10^5^ Raji Luc^+^ cells. After 7 days, 1 × 10^6^ CAR T cells or Mock GFP-transduced T cells, adjusted to the total amount of T cells according to transduction efficiency of the CARs, were infused intravenously.

For AsPC1 GFP^+^/Luc^+^ cell line derived tumors 1 × 10^6^ cells were injected subcutaneously in the right flank of NOD SCID gamma (NSG; NOD.Cg-PrkdcscidIl2rgtm1Wjl/SzJ) mice (Jackson Laboratory, provided by Charles River). When tumors reached a size of 25 mm^2^, 5 × 10^6^ CAR T cells were injected into the tail vein. The amount of injected untransduced Mock T cells was adjusted to the number of total T cells in the CAR group with the highest total cell count.

Therapeutic response was measured longitudinally using the IVIS Lumina *in vivo* imaging system (PerkinElmer) after intraperitoneal injection of 100 μL (30 mg/mL) D-Luciferin (for Raji studies: XenoLight Rediject D-Luciferin Ultra (PerkinElmer). For AsPc1 studies: Potassium Salt, LUCK-2G, GoldBio) and additionally by manual caliper measurement for pancreatic tumors. All measures to secure the well-being of mice were executed following the relevant animal use guidelines and ethical regulations. Upon reaching the endpoint (weight loss of >19%, paralysis, stress score of >20 or end-point of the experiment, Day 20 for the lymphoma model and Day 29 for the pancreatic model), animals were euthanized according to guidelines and post-mortem analysis was performed in order to determine tumor burden, persistence and killing of the different CAR T cell constructs. In particular blood, bone marrow and spleen were subjected to flow cytometric analysis. Therefore, spleen was dissociated using the gentleMACS™ Octo Dissociator with Heaters according to the manufacturers protocol (Miltenyi Biotec) and bone marrow was extracted from the femurs and tibias of mice by cutting off the epiphyses of the bones and rinsing the inner fragments. The cell suspensions were filtered through a 70 μm pore size MACS SmartStrainer (Miltenyi Biotec) and following red blood cell lysis on blood, bone marrow and spleen single cell suspensions using Red Blood Cell Lysis Solution (Miltenyi Biotec), samples were stained and analysis was conducted on a MACSQuant Analyzer 8.

### Statistics

Unless otherwise specified, all graphical error bars represent standard error of the mean. Statistical comparisons between more than two groups were conducted by One-way ANOVA with *p* < 0.05 using GraphPad Prism 7. To facilitate the statistical overview of the *in vivo* experiments, the significance analyses were organized in a pairwise significance matrix (PSM) where each box represents a comparison between two groups, as shown by Al Rawashdeh et al. (40). The order, in which the groups were compared, is illustrated in Figures S1, S4. Significant differences between two comparing groups are defined by a green box, while insignificant differences by a red box.

## Results

### CD20 Specific CD8α and IgG1 CH2-CH3 Spacer CARs Exhibit Comparable *in vitro* Activity

**Figure 1:**
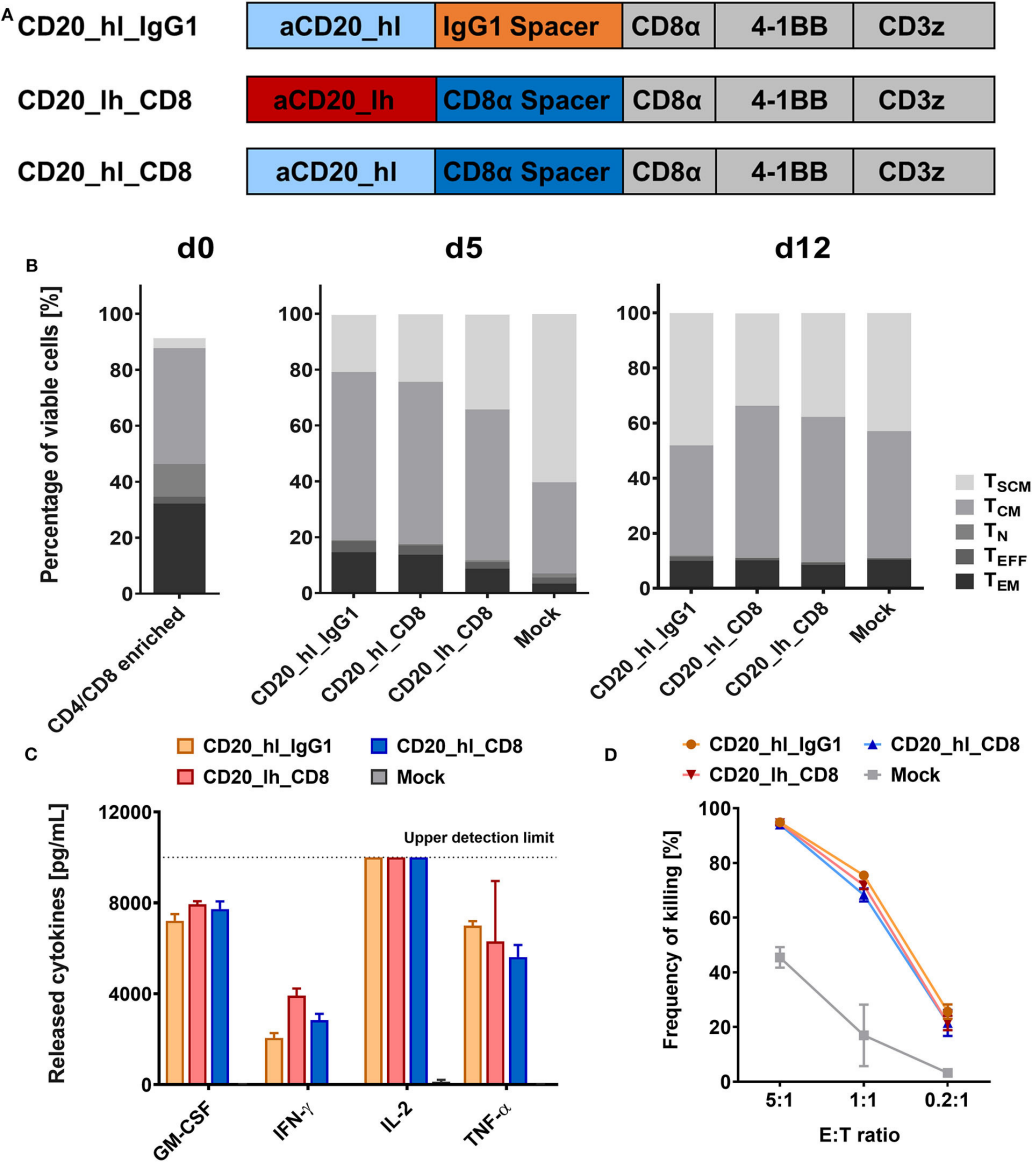
CD20 specific CAR T cells with short CD8α and long IgG1 CH2-CH3 spacers show similar *in vitro* functionality. **(A)** Structure of the three CD20 CAR constructs. **(B)** T cell phenotypes in the CD4/CD8 enriched fraction on d0, d5, and d12 of the automated T cell transduction process by flow cytometry. **(C)** GM-CSF, IFN-γ, IL-2, IL-6, and TNF-α production after 24 h co-culture of CD20 CAR T cells with CD20^+^ JeKo-1 target cells at 1:1 effector to target ratio analyzed by flow cytometry. *n* = 3. **(D)** Cytolytic activity of the engineered CAR T cells. Effector CAR T cells and target-positive JeKo-1 target cells were co-cultured for 24 h at the indicated ratios (E:T). Detection of the specific lysis was performed by flow cytometry. *n* = 3.

During pre-clinical development of a CD20 directed CAR candidate (39) we also evaluated a number of different CAR configurations (Figure 1A). We used an scFv derived from the Leu16 monoclonal antibody (30, 41), binding to the large extracellular loop of CD20 (42). This loop is only 47 amino acids long, which is why we hypothesized it would be more effectively targeted with a flexible CD8α or long IgG spacer. We generated two second generation CAR constructs, that comprised a CD8α transmembrane domain, a 4-1BB co-stimulatory domain and a CD3ζ main activator domain. Both bind CD20 via the Leu16 derived scFv in a V_H_-V_L_ orientation and only differed in the spacer domain. The CD20_hl_IgG1 CAR comprises an IgG1 CH2-CH3 spacer while the CD20_hl_CD8 CAR possesses a CD8α spacer. The PELLGG and ISR motif of the IgG1 CH2-CH3 spacer were replaced by the corresponding IgG2 amino acids to reduce Fcγ-Receptor binding, as described previously (23). To assess whether the order of binding domains in the scFv also can play a role in CAR function, we constructed a CD8α spacer CAR with swapped scFv orientation (CD20_lh_CD8). We generated CD20 specific CAR T cells by genetically modifying CD3/CD28 polyclonally activated T cells with lentiviral vectors in a fully automated manner in a closed system using the CliniMACS Prodigy® as described previously (39). At the end of the manufacturing on day 12, similar T cell phenotypes were obtained for the samples modified with the different CAR constructs and the untransduced Mock control (Figure 1B). More than 80% of T cells had a memory phenotype (central memory T cell (T_CM_) and stem cell memory T cell (T_SCM_) as defined by their phenotypes being CD62L^+^/CD45RO^+^/CD95^+^ and CD62L^+^/CD45RO^−^/CD95^+^, respectively). Also, the three constructs demonstrated comparable functionality in terms of cytokine release (Figure 1C) and cytotoxicity (Figure 1D) upon co-culture with CD20^+^ JeKo-1 target cells.

### CD8α and IgG1 CH2-CH3 Spacer CARs Differ in Their *in vivo* Performance

**Figure 2:**
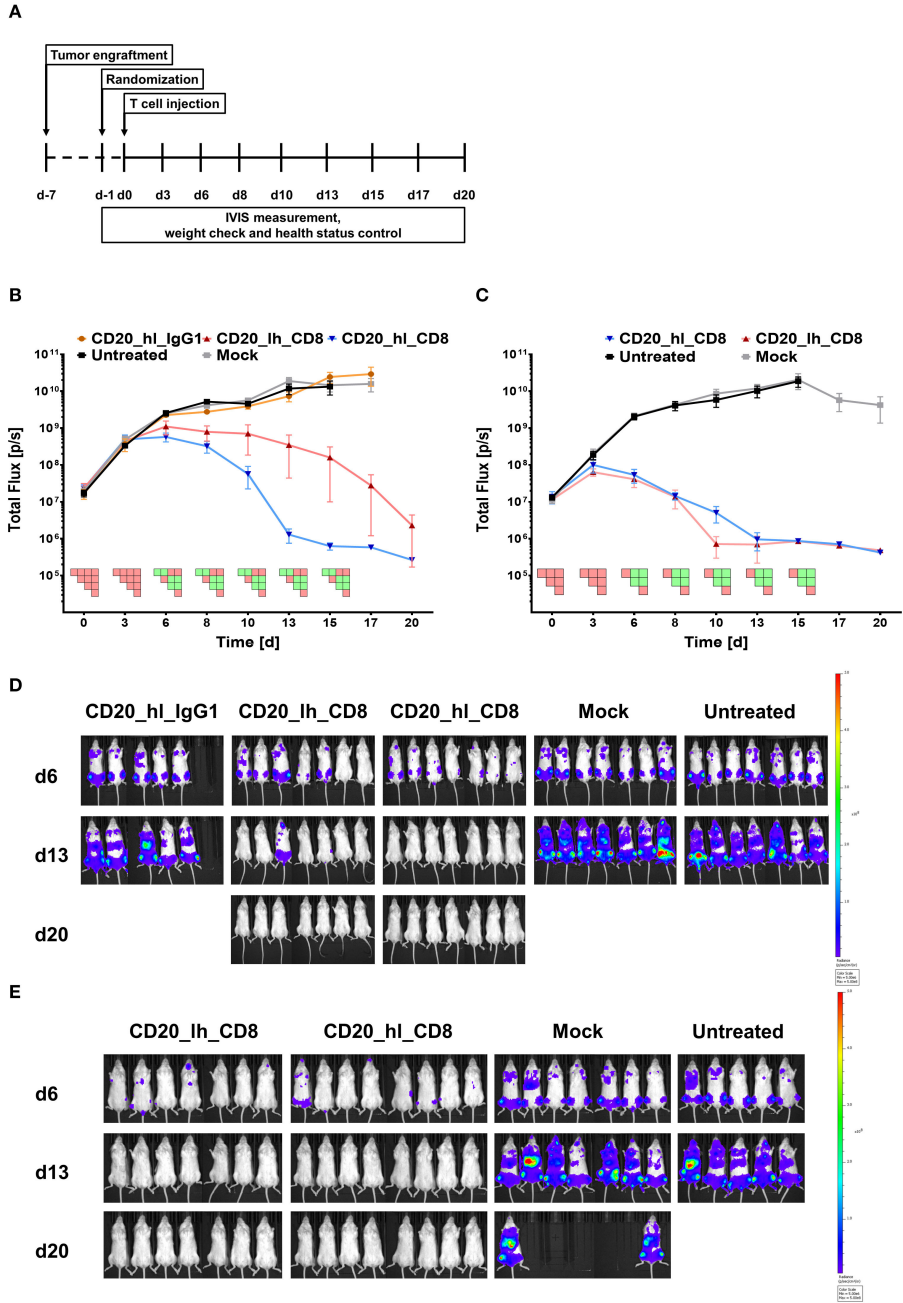
CD20 specific CAR T cells with an IgG1 spacer domain fail to exhibit *in vivo* efficacy. **(A)** Overview of the study workflows. **(B)** Tumor burden change over time in mice treated with anti-CD20 IgG1 CH2-CH3 and CD8α spacer CAR T cells from one donor. *n* = 5/group. PSM *p* < 0.05 (green) [one-way ANOVA]. **(C)** Tumor burden change over time in presence of the two different CD8α CAR constructs with T cells from a second donor. *n* = 6/group. PSM *p* < 0.05 (green) [one-way ANOVA]. **(D)** Representative *in vivo* bioluminescence images of tumor bearing mice from **(B)**. Images are arranged according to the treatment group and time after CAR T cell injection. T cells were generated from one donor. Scale factor: min: 5 × 10^6^, max: 5 × 10^8^ p/s. **(E)** Representative *in vivo* bioluminescence images of tumor bearing mice from **(C)**. Images are arranged according to the treatment group and time after CAR T cell injection. T cells were generated from a second donor. Scale factor: min: 5 × 10^6^, max: 5 × 10^8^ p/s.

Having assessed the *in vitro* activity, we next analyzed the same lentivirally modified T cells in a pre-clinical NSG mouse model. 5 × 10^5^ CD20^+^ Raji cells, which were modified to constitutively express luciferase, were injected into the tail vein of each mouse. Seven days later, 1 × 10^6^ CD20 specific CAR T cells or GFP transduced Mock T cells (Figure 2A) were also applied intravenously. Tumor burden was monitored longitudinally over 3 weeks by non-invasive bioluminescent imaging (BLI) of tumor cells *in vivo*. Neither the Mock treated group nor mice treated with the IgG1 spacer CAR showed any control of tumor growth compared to the untreated group, and the animals in these groups were sacrificed according to the ethical code on day 17 and day 15, respectively (Figure 2B). On the other hand, significant therapeutic responses were achieved by the CD20_hl_CD8 and CD20_lh_CD8 CAR T cells (Figures 2B,D). Both groups exhibited a reduced tumor growth 6 days post T cell injection. While the CD20_hl_CD8 CAR T cells reached background fluorescence on day 13, CAR T cells equipped with the same CAR but with the scFv in the converse orientation needed longer to reduce tumor burden and did not reach background levels until the end of the experiment. This difference between the scFv variants could be attributed to a single mouse having remnants of tumor present in the jawbone, which in our experience is difficult to treat and possibly inaccessible to CAR T cells. We verified that the scFv orientation indeed had only a minor influence by repeating the experiment with the CD8α spacer CARs with the different scFv orientations using a different donor (Figures 2C,E). Again, both groups of CAR-modified T cells were effective in rapidly controlling the tumor growth, with no significant difference being observed between the different scFv orientations. *Ex* vivo analysis of spleen, bone marrow and blood at the end of the study showed no detectable IgG1 spacer CAR T cells in the treated mice while CAR T cells with the CD8α spacer could be readily detected, implying a reduced *in vivo* persistence or expansion of the T cells modified with the IgG1 spacer CAR (Figure S2).

These findings were in line with earlier results of other groups, showing reduced *in vivo* efficacy of full length IgG family spacers (25, 27). These groups mutated FcR binding sites or developed other solutions in order to decrease off-target binding of the T cell, which we were also able to confirm in an *in vitro* assay using mouse macrophages (Figure S3), but it is unclear whether all potential off-target binding has been abrogated as binding to other lower affinity FcγRs may be retained (25).

### Construction and Characterization of a New Family of CAR Spacers

**Figure 3:**
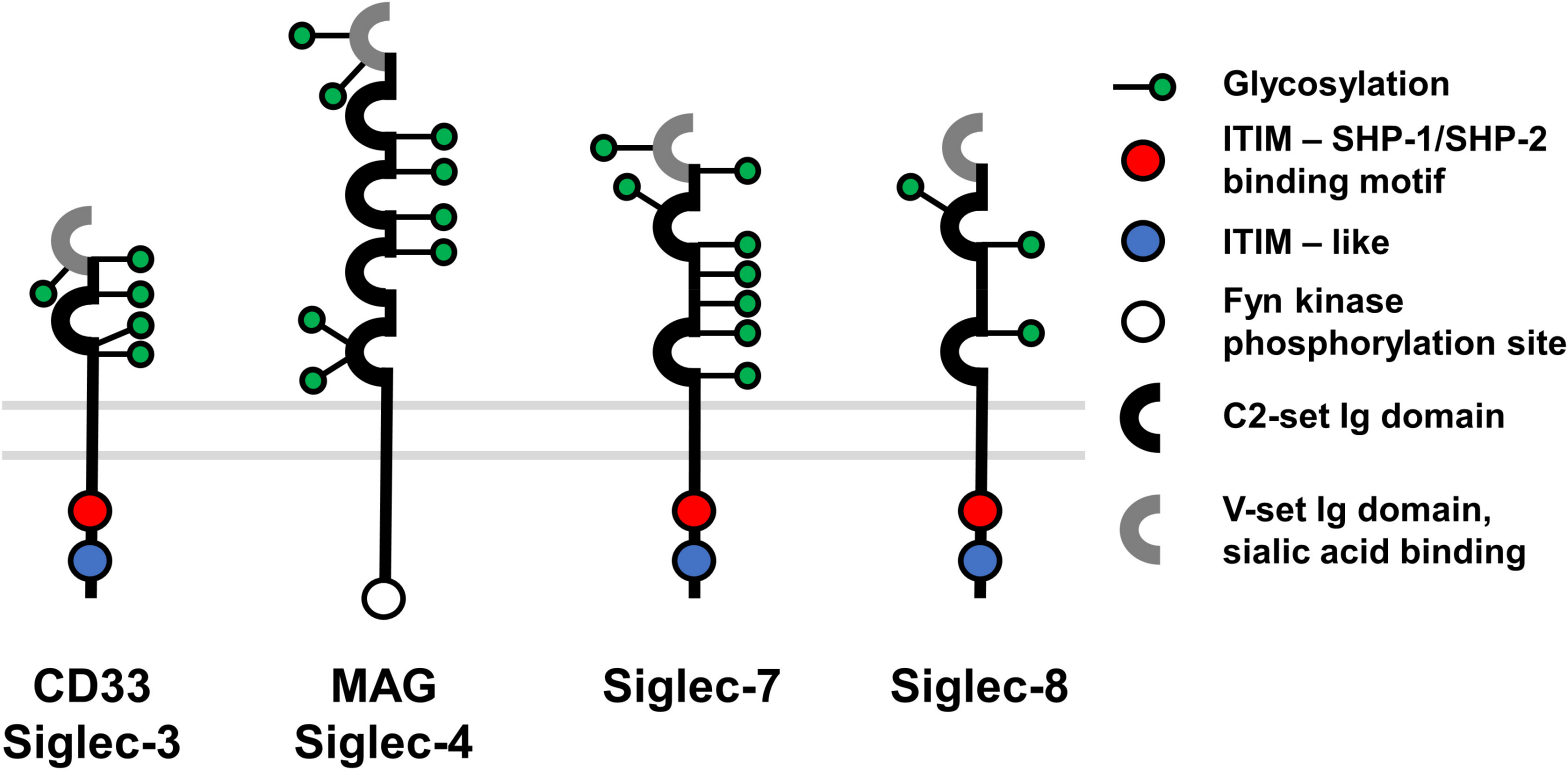
Overview of Siglec membrane proteins used for CAR construction.

These findings motivated us to develop a new class of CAR spacer regions that naturally lack FcR binding sites. In this context we identified the Sialic acid-binding immunoglobulin-type lectin (Siglec) family whose members are expressed on various immune cells and incorporate Ig-like domains in their receptor architecture (43, 44). More specifically, while the membrane distal sialic acid binding Ig-like V-set domain is positioned N-terminally, the more C-terminally located Ig-like C2-set domains, which vary in number, serve as spacer regions. Based on previous reports describing that CAR T cell activation can be optimized according to the epitope location and spacer length, we selected one, two or three C2-set domains derived from Siglec-3, -4, -7, or -8 for spacer design (Figure 3).

**Figure 4:**
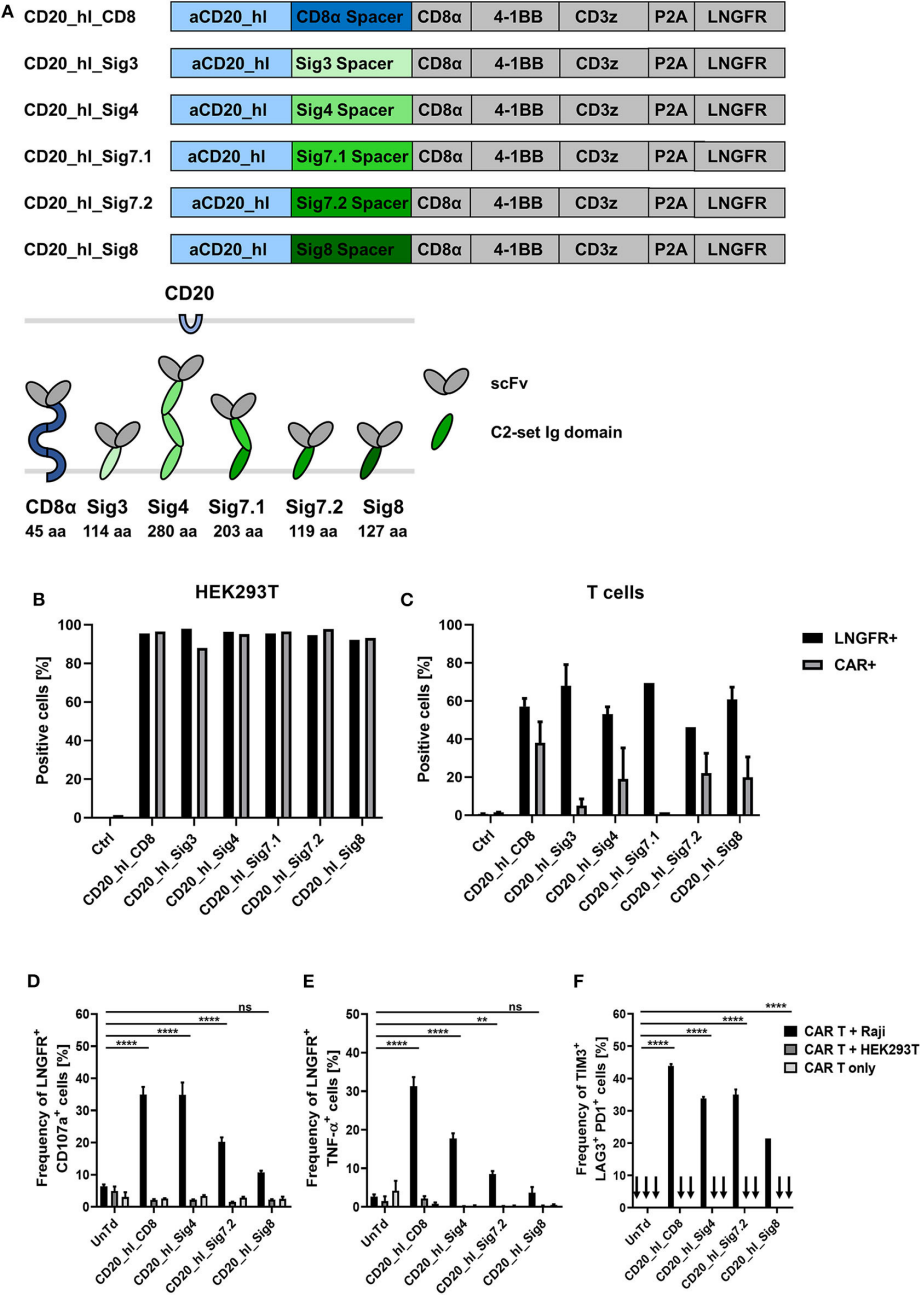
*In vitro* evaluation of novel CD20 specific Siglec spacer CAR T cells. **(A)** Modular structure of the CD20 CAR constructs with the Siglec spacers and extracellular domain comparison of the CAR constructs. **(B)** Expression analysis of the CAR constructs in transiently transfected HEK293T cells 24 h post transfection and **(C)** in transduced T cells from two donors 6 days post transduction. LNGFR and CAR expression were evaluated by flow cytometry. **(D,E)** Siglec spacer CAR T cells were cocultured with Raji or HEK293T cells for 5 h at a ratio of 1:1 and T cell expression of CD107a **(D)** and intracellular TNF-α **(E)** were analyzed by flow cytometry. **(F)** The frequency of TIM3, LAG3, and PD1 triple positive CAR T cells was analyzed after 24 h co-culture at a 1:2 ratio of CAR T cells to Raji or HEK293T cells by flow cytometry. CAR T cells alone were also cultured in order to exclude unspecific activation. *n* = 3. Error bars, mean ± SD. *ns* > 0.05, **p < 0.01, and ****p < 0.0001 [one-way ANOVA, CAR T + Raji compared to Untreated (UnTd)].

To confirm correct translation and surface expression of the constructs, bicistronic lentiviral expression vectors were generated with a downstream ΔLNGFR gene linked to the CAR by a P2A sequence (Figure 4A). After transfection of the DNA constructs into HEK293T cells, detection of the reporter protein ΔLNGFR confirmed successful transcription and translation of the CAR cassette, while direct staining of the CAR with a CD20 CAR detection reagent (PE) visualized surface expression of the CAR constructs. All constructs showed both ΔLNGFR and CAR expression in >80% of HEK293T cells (Figure 4B). Subsequently, we transduced primary T cells with lentiviral vectors and assessed the CAR expression 6 days post transduction (Figure 4C). The ΔLNGFR reporter protein was expressed in all cases demonstrating effective lentiviral transduction of the T cells and translation of the expression cassette (range 46–75% LNGFR^+^ T cells). However, while three CAR constructs showed CAR expression levels comparable to the CD8α spacer CAR control, no CD20_hl_Sig7.1 CAR expression was detectable and the CD20_hl_Sig3 CAR was expressed on only 5% of the T cells. Based on these results, we excluded these constructs from further analysis.

### Siglec-4 Spacer Shows Comparable *in vitro* Functionality To CD8α Spacer in a CD20 CAR Model

Next, we investigated the cytotoxic potential of the novel constructs. We co-cultured CAR T cells for 5 h with CD20^+^ Raji cells or CD20^−^ HEK293T cells at an E:T ratio of 1:1. Effector function was assessed by determining degranulation and intracellular detection of the cytokine TNF-α in the transduced cells (gated on ΔLNGFR expression). Only CAR T cells co-cultured with CD20^+^ target cells showed significant degranulation (Figure 4D). Strongest degranulation could be observed for the CD8α and Siglec-4 spacer variants with around 35% of CD107α positive cells. The Siglec-7.2 spacer CAR produced an intermediate amount of CD107α at 20% positive cells and the Siglec-8 variant had the lowest degranulation with only 10% positive cells but still more than the negative controls (Figure 4D). Similar to the degranulation analysis, the proportion of ΔLNGFR^+^/TNF-α^+^ cells was also highest in CD8α spacer CAR T cells (Figure 4E; 31%) but the CD20_hl_Sig4 CARs only displayed 18% of TNF-α positive cells, followed by Siglec-7.2 and Siglec-8 spacer CARs. Again, no unspecific activation could be observed in the controls.

We also assessed the activation state of the modified T cells by analyzing TIM3, LAG3, and PD1 surface expression. CD20^+^ Raji cells were co-cultured with CAR T cells for 24 h at an E:T ratio of 1:2. The CD8α and Siglec-4 spacer CAR modified T cells contained the largest fraction of TIM3/LAG3/PD1 triple positive cells (Figure 4F). As the Siglec-7.2 and Siglec-8 spacer CAR T cells displayed lower degranulation and upregulation of activation markers after antigen engagement throughout these *in vitro* experiments, we decided to investigate only the Siglec-4 spacer in more detail.

### The Siglec-4 Spacer CAR Displays High Functional Potency Against Membrane-Proximal Targets

In our CD20^+^ Raji lymphoma model the Siglec-4 spacer CAR demonstrated a comparable *in vitro* functionality to the CD8α spacer CAR. As described above the Leu16 epitope is very proximal to the target cell membrane, making it more susceptible for engagement with long spacer CARs. From the CAR variants that could be efficiently expressed in T cells, the Siglec-4 spacer was the only spacer with three C2-set Ig domains, agreeing with previous work that long spacers are excellent for targeting “short,” membrane-proximal targets. To verify this hypothesis and to prove the robustness of the Siglec-4 spacer functionality, we assessed the Siglec-4 spacer CAR in an additional solid tumor model of pancreatic ductal adenocarcinoma (PDAC). We have recently identified CD66c and TSPAN8 as novel target candidates for cellular treatment of PDAC (Schäfer et al. manuscript under revision). These two target molecules are especially suitable for investigating our novel long spacer, as the scFv binding epitopes differ greatly in terms of membrane proximity.

TSPAN8 has two extracellular loops extending from the membrane that span 24 and 96 amino acids, respectively, the larger having two interconnecting disulfide bonds. Thus, the whole protein is very membrane proximal. On the other hand, CD66c is a glycophosphatidylinositol anchored protein and consists of two C2-set domains and one V-set domain. In consequence it extends further into the extracellular space compared to TSPAN8. In addition, the epitope of the aCD66c scFv is localized on the outer N terminal V-set domain. In summary, TSPAN8 can be considered a membrane proximal target, while CD66c is a membrane distal target.

**Figure 5:**
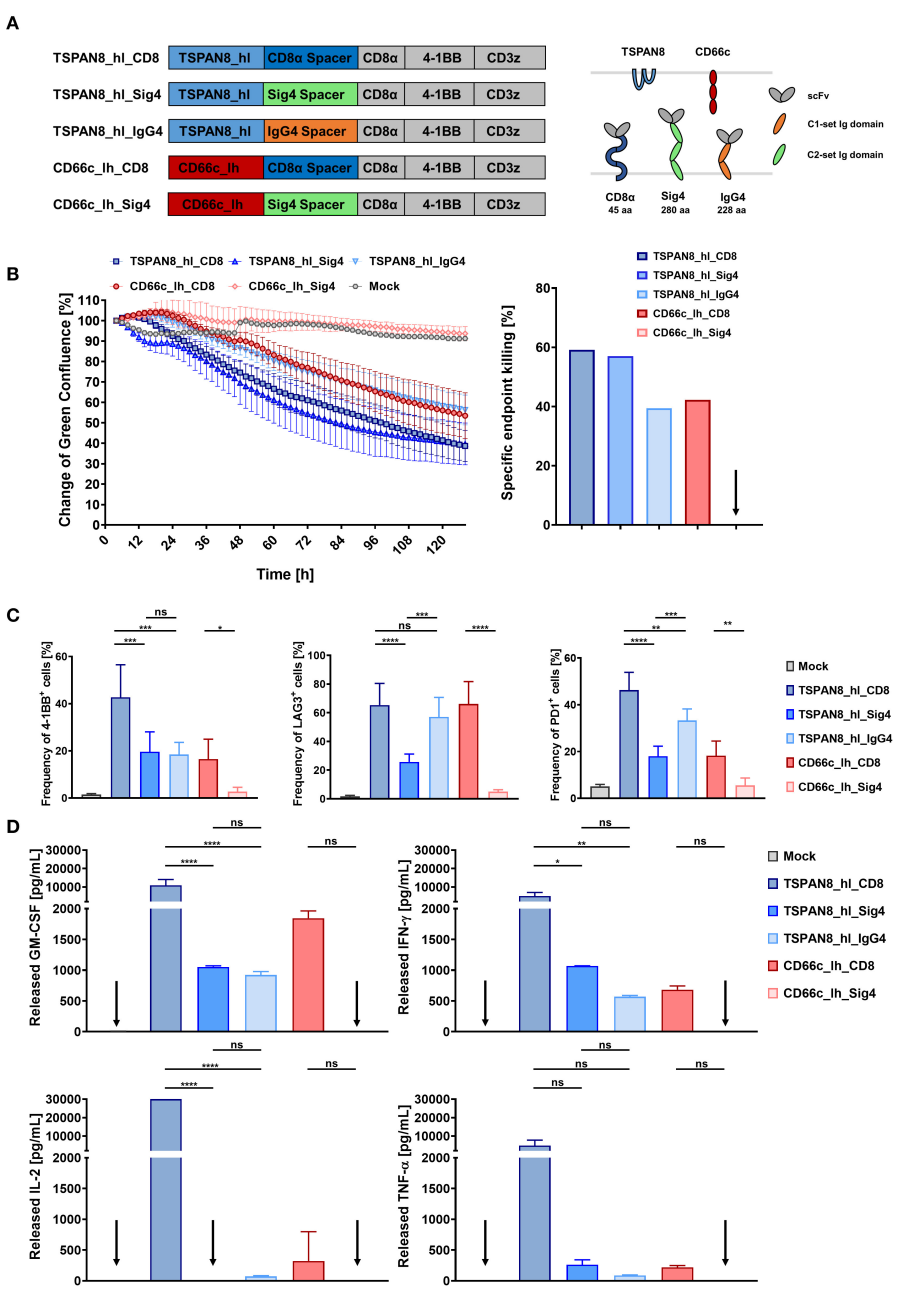
*In vitro* comparison of T cells transduced with TSPAN8 and CD66c CAR constructs, incorporating different spacer domains. **(A)** Structure of the TSPAN8 and CD66c CAR constructs with the Siglec spacers and extracellular domain comparison of the CAR constructs and target molecules. **(B)** Cytolytic kinetics and specific endpoint killing of AsPC1 target cells incubated with CAR T cells and Mock T cells from three different donors in effector to target ratios of 2:1. *n* = 6. **(C)** Frequency of 4-1BB, LAG3 and PD1 positive CAR T cells was analyzed at the end of the cytolytic evaluation with AsPC1 cells by flow cytometry. **(D)** GM-CSF, IFN-γ, IL-2, IL-6, and TNF-α production after 24 h of co-culture of TSPAN8 or CD66c CAR T cells with AsPC1 cells from one donor assessed by flow cytometry. *n* = 2. Data from **(B–D)** were taken from the same experiment. Shown is the mean ± SD. *ns* > 0.05, *p < 0.05, **p < 0.01, ***p < 0.001, and ****p < 0.0001 [one-way ANOVA, multiple comparisons].

We exchanged the Leu16 scFv from our CD20_hl_Sig4 CAR with the CD66c and TSPAN8 specific scFvs that were previously identified (Figure 5A) (Schäfer et al. manuscript under revision). Additionally, we incorporated in our experiments CD66c and TSPAN8 specific CD8α spacer CARs and a TSPAN8 specific IgG4 CH2-CH3 spacer CAR, which contained a 4/2 NQ mutation in the CH2 domain as well as a S → P substitution which has been reported to reduce FcR binding also *in vivo* (25), which was not the case for our IgG1 construct (25).

CD66c^+^/TSPAN8^+^ AsPC1 PDAC cells that were additionally modified to express GFP and luciferase were co-cultivated with CAR T cells specific for CD66c and TSPAN8 at an E:T ratio of 2:1 and analyzed using a fluorescent live cell analysis system. We assessed cytotoxicity as a decrease in green fluorescence surface area normalized to 2 h after co-inoculation. After 48 h, a supernatant sample was taken for cytokine quantitation while activation markers were measured at the end of the experiment (132 h).

Both, the CD66c_lh_Siglec-4 CAR T cells, as well as the untransduced control T cells showed no specific killing of target cells, while the CD66c_lh_CD8 CAR showed a specific endpoint killing of 42%, (Figure 5B). On the other hand, when targeting the membrane proximal TSPAN8, the Siglec-4 spacer CAR T cells showed a similar killing to that of the TSPAN8_hl_CD8α CAR T cells approaching 60% endpoint killing. In contrast, CAR T cells modified with a TSPAN8 CAR with the alternative long IgG4 CH2-CH3 spacer exhibited only 40% killing at the end of the experiment, showing the weakest cytotoxicity of all tested TSPAN8 CAR T cells. The CD66c_lh_Sig4 CAR T cells, which showed no cytotoxicity, also expressed no activation markers (Figure 5C). The strongest upregulation of activation markers 4-1BB, LAG3 and PD-1 was observed in TSPAN8_hl_CD8α CAR T cells. Interestingly, the TSPAN8 specific Siglec-4 CAR T cells displayed a lower expression of activation markers, even though the cytotoxicity equalled that of the CD8α spacer CAR T cells. This difference between the CD8α and the Siglec-4 spacer CAR T cells was even more striking at the cytokine level (Figure 5D). The TSPAN8_hl_CD8 CAR T cells released markedly higher levels of cytokines than the other CAR T cells. The TSPAN8_hl_Sig4 CAR T cells secreted cytokines at levels more similar to CD66c_lh_CD8 and TSPAN8_hl_IgG4 CAR T cells, which was very surprising, with regard to the same observed cytotoxicity as the TSPAN8 CD8α CAR T cells.

### The Siglec-4 Spacer Is Highly Efficacious in an *in vivo* PDAC Model

**Figure 6:**
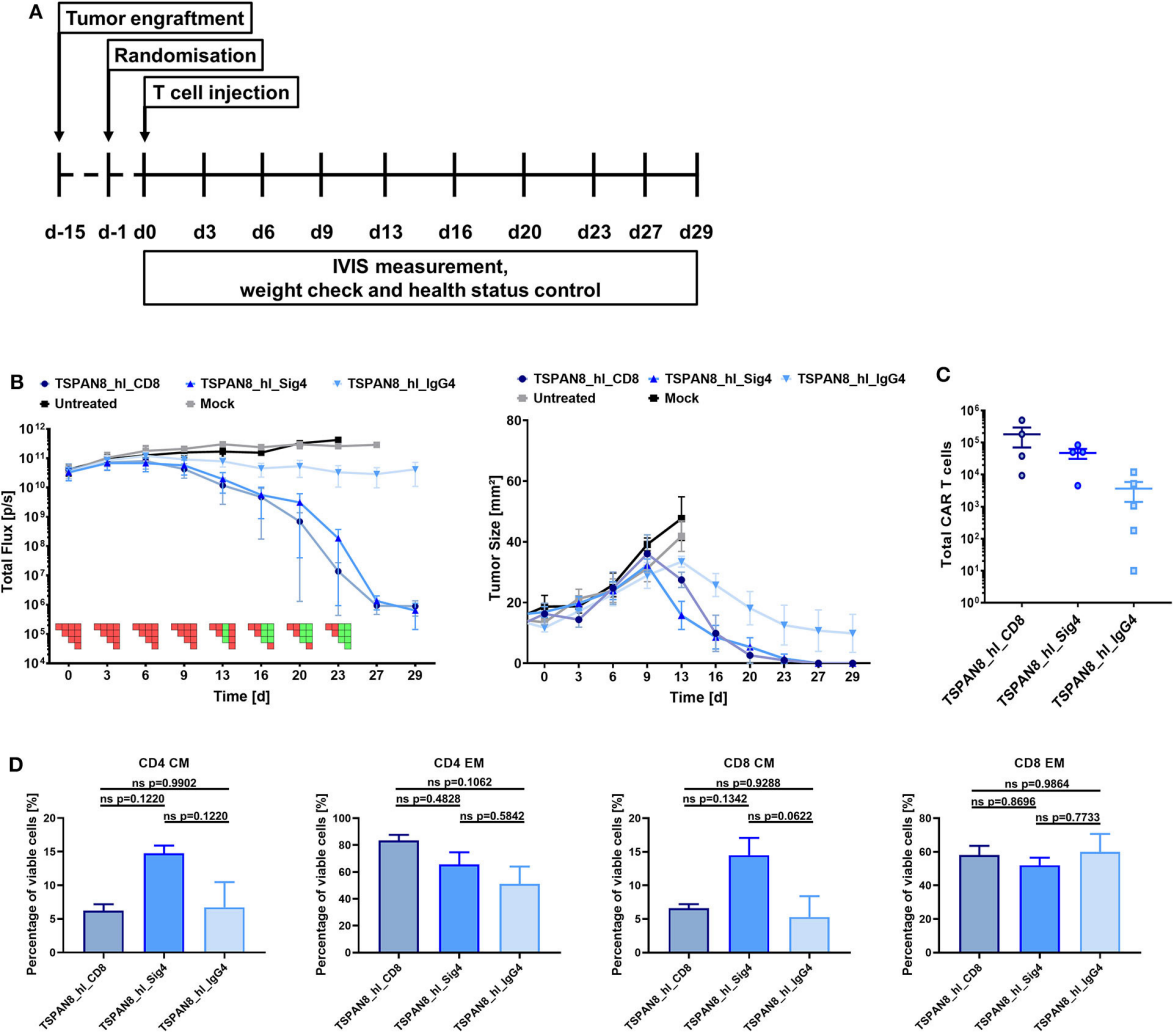
The TSPAN8 specific Siglec-4 spacer CAR T cells exhibit the same anti-tumor efficacy as the CD8α spacer CAR T cells, while retaining a more memory-like phenotype. **(A)** Overview of the study workflow. **(B)** Tumor burden and change in tumor size over time after TSPAN8 CAR T cell infusion. Untreated and Mock T cell treated animals served as controls, T cells from one donor were used. IgG4: *n* = 5; Sig4 and CD8α: *n* = 4. PSM *p* < 0.05 (green) [one-way ANOVA, multiple comparisons]. **(C)** Total number of CAR positive T cells recovered from spleens of TSPAN8 CAR-treated animals at the end of the experiment calculated after flow cytometric analysis. IgG4: *n* = 5; Sig4 and CD8α: *n* = 4. **(D)** CD4 and CD8 CAR+ T cell phenotypes in the spleens of TSPAN8 CAR-treated animals analyzed at the end of the experiment by flow cytometry. *n* = 4.

Finally, we investigated the functionality of the three TSPAN8 specific CAR constructs *in vivo* in a pre-clinical PDAC tumor model. 1 × 10^6^ AsPC1 eGFP^+^/Luc^+^ cells were injected subcutaneously in NSG mice. Tumor growth was measured non-invasively by BLI imaging and furthermore assessed by physical caliper measurement. When the first tumors reached a diameter of 25 mm^2^, treatment groups were randomized according to the BLI signal and tumor size, and treatment was started by i.v. injection of 5 × 10^6^ CAR T or untransduced Mock T cells (Figure 6A). Untransduced T cells did not display a therapeutic benefit over the untreated group (Figure 6B). All mice from these two groups had to be sacrificed before the end of the experiment as tumor ulcerations began to become established. The therapeutic effect for the CD8α and Siglec-4 CARs became apparent in BLI measurements from day 6 onwards. The tumor burden within the groups treated with the CD8α and Siglec-4 spacer CARs decreased in a comparable manner and reached baseline by the end of the experiment 29 days after T cell injection. At the same time, tumor growth was controlled by the IgG4 CH2-CH3 spacer group, but there was no tumor clearance as seen with the other groups. Persistence of CAR T cells could be demonstrated in the spleens of all CAR T cell treated groups with the highest amounts found in the CD8α spacer CAR and Siglec-4 spacer CAR groups (Figure 6C). A markedly lower amount of CAR T cells could be recovered from the IgG4 spacer CAR group. Interestingly, when the phenotype of the human T cells was examined the proportion of T_CM_ was twice as high in CD4 and CD8 CAR T cells of the Siglec-4 spacer CAR group as compared to the CD8α spacer CAR T cells (Figure 6D).

## Discussion

Despite the largely empirical design of CARs based on the functional principles of an antibody and the T cell receptor (TCR), CAR T cell therapies have demonstrated remarkable efficacy in the hematological tumor setting. Although a direct comparison of results across CAR T cell-based clinical trials is difficult due to the various differences in protocols, target antigens, co-stimulatory signaling, treatment regimen, patient groups and disease burden, the rough trend can be observed that those receptors that incorporate a CD8α or CD28 spacer region in their architecture display better therapeutical efficacy than those that utilize IgG-based Fc domains (1–7, 28–31). Non-clinical studies investigating this effect suggest that the inferiority of IgG spacers is due to the engagement with FcγR-expressing myeloid cells (23) resulting in off-target activation of both gene-modified T cells and the respective FcγR^+^ cells. In parallel, additional work has been demonstrating that the exemplary performance of CD8α or CD28 spacer CARs is partially also attributed to the epitope location on the targeted antigen CD19 and a number of studies have affirmed the postulate that membrane-proximal epitopes are best targeted by long spacer modules while membrane-distal epitopes are effectively recognized by CARs incorporating short spacer elements (18–22, 45).

In light of these developments, we identified a shortage of functional CAR spacer modules for membrane-proximal epitopes. Taking advantage of the well-described CD20 antigen and the membrane-proximal binding epitope of Leu16-derived anti-CD20 scFv (42), we sought to characterize the properties of CD8α- vs. IgG-based spacer CARs against CD20 *in vivo*. To avoid unintended cross-activation of CAR- and FcγR-expressing cells in the context of the IgG spacer, the amino acid sequence for IgG1-FcγR interactions in the IgG1-CH2 extracellular domain of the CAR was replaced by the corresponding IgG2 amino acids as described previously (23). However, contrary to reports describing increased anti-tumor activity and CAR T cell persistence following modifications in the IgG4 spacer to abrogate FcγR-binding in the CAR spacer domain (25, 27), we did not observe any *in vivo* therapeutic efficacy of IgG1 CAR T cells after similar modifications in our study. More specifically, the lack of efficacy was accompanied with an inefficient persistence of the gene-modified T cells. These results were in stark contrast to the functional capacity of the CD8α CAR T cells which – according to current understanding – display a less favorable receptor architecture due to the short spacer region. Although it is reasonable to conclude that the introduced mutations into the IgG spacer domain may not entirely abrogate FcγR binding, it cannot be ruled out that additional mechanisms are in play that sacrifice the therapeutic efficacy. For example, it has already been described that murine scFvs and other non-self gene products can elicit HLA-restricted T cell-mediated immune responses (3, 46, 47). Thus, the possibility exists that the introduced mutations into the Fc region can create immunogenic peptides by the T cell's antigen processing machinery which are then presented on the T cell's HLA and render the gene-modified lymphocytes susceptible to TCR-triggered fratricidal activity. Therefore, it is to be appreciated that the interplay of CAR T cells with their cognate counterparts and the immune system is complex and further work is required to understand the full immunogenic potential of CAR molecules.

To exclude the possibility of potential immunological barriers elicited by the spacer region, we switched our test system to the IgG4 backbone which was previously described to show *in vivo* performance (25, 27) and which has also shown successful translation to the clinic (34). In addition, a new set of spacer domains was designed based on the Siglec family whose members are expressed throughout the immune system and display evolutionary structural similarities to the constant region of immunoglobulins, but lack the inherent ability to interact with FcγRs (35, 36). To determine systematically the optimal spacer length for the membrane-proximal CD20 epitope, five Siglec spacer CAR variants were generated incorporating either one, two or three Ig domains. Of note, different parent proteins were selected, as different Siglec molecules encompass distinct glycosylation patterns which are likely involved in modulating the protein's stability, flexibility, spatial architecture etc. and thus may have different effects on the CAR molecule. Moreover, in an attempt to maintain the original architecture of the molecule, the domains closest to the plasma membrane were selected. Consequently, the Siglec spacer regions within the otherwise identical CAR framework encompassed either a 114 amino acid (aa) Siglec-3, 119 aa Siglec-7, 127 aa Siglec-8, 203 aa Siglec-7, and 280 aa Siglec-4 spacer domain as opposed to the control 45 aa CD8α spacer domain.

Subsequent expression profiling revealed that not all Siglec spacer-based CARs were efficiently expressed on the T cell surface. In particular, Siglec-7.1 and Siglec-3 spacer CARs showed the lowest expression efficiency emphasizing the importance of the spacer region not only on the receptor's functionality but also on its optimal expression. In fact, Patel and colleagues have already described that the CAR spacer domain can affect the receptor's stability and modify its turnover rate (17). It is plausible that the glycosylation patterns present in Siglec-7.1 and Siglec-3 spacer CARs render the receptors less stable, in this way increasing the turnover kinetics and a decreased CAR detectability on the cell surface. Another potential reason for the inefficient expression of the Siglec-3 spacer CAR may lie in the C169S mutation which was introduced in order to abrogate unspecific disulfide bond formation as C169 is involved in an interdomain disulfide bond within the parent protein. Moreover, it is possible that the Siglec-3 C2-set domain *per-se* is instable when isolated from the membrane-distal V-set domain. Although a splice variant of CD33 has been described, which lacks the N-terminal domain (CD33^Δ*E*2^), these reports rely on mRNA analyses (48, 49). Protein-based detection using antibodies remains controversial, as it is still not clear whether a clone exists that can specifically recognize the Siglec-3 C2-set domain (49, 50). Importantly, using lentiviral transduction of His-tagged CD33^Δ*E*2^, Laszlo and colleagues have shown that the expression of the splice variant is also dependent on the cell type (49). In this context, HEK293T exhibited highest transgene expression while hematopoietic cells displayed only low level expression of the truncated immune receptor which is in line with our observations on the expression of the Siglec-3 spacer CAR (Figures 4B,C).

In the next series of experiments, the three best expressed Siglec spacer CAR candidates were analyzed for their ability to induce T cell effector function upon antigen engagement. Consistent with previous reports (17–19, 25, 26, 51), our study provides evidence that the CAR spacer region can modulate the effector function of transgenic T cells. Intriguingly, however, we find that depending on the effector function analyzed, the functional hierarchy may vary. In particular with regard to cytotoxicity, no significant differences between the CD8α spacer (45 aa in length, no Ig domain) and Siglec-4 spacer (280 aa in length; three Ig domains) CAR can be observed while in terms of cytokine secretion the CD8α spacer CAR displays a significant dominance over other CAR constructs. Importantly, in addition to the CD20 system, this observation was further confirmed in the setting of another membrane-proximal antigen, TSPAN8, indicating a common functional feature for membrane-adjacent epitopes.

It has already been demonstrated in the TCR-context that distinct thresholds exist for the cytolytic machinery, the proliferative induction as well as the cytokine production system (52–56) and emerging work suggests similar principles for CAR-triggered T cells (26). The current study further supports this finding and the data obtained indicate that the nature of the spacer region can modulate the nature and degree of effector function. An alternative strategy has been described by Liu and colleagues (57) and Caruso and colleagues (58) in two independent studies, in which they demonstrate the ability of effector function fine-tuning through scFv affinity modulation. The clinical impact of such modifications was impressively demonstrated by Ghorashian and colleagues, who reported a better overall therapeutic profile of CD19 CAR T cell therapies in patients who received lower affinity CARs compared to the commonly used FMC63-scFv-based CARs (59). In particular, while the antileukemic activity was retained, the CAR T cells displayed an enhanced proliferative capacity and reduced severity of cytokine release syndrome (CRS). Though this clearly reveals the effectiveness of such an approach, scFv affinity modulation is a laborious undertaking and bears the risk to result in unwanted modifications to the target specificity. Therefore, fine tuning the chimeric receptor's spacer region provides a time-profitable option with a lower risk profile. More importantly, it further allows to create a variety of receptors with a range of signal transduction intensities independent of the binding domain.

Besides, based on the efficacy data obtained with the CD8α spacer (45 aa) vs. Siglec-4 spacer (280 aa) CARs targeting CD20 and TSPAN8, we find that the receptors' cytotoxic efficacy is not dominated by the spatial constraints of the CAR and its target epitope. This is significant as previous studies reporting such a trend were performed primarily in the context of IgG-derived sequences (25, 26, 51) and have not been compared extensively to spacers derived from other parental proteins. Thus, our work demonstrates that not only structural and spatial elements in CAR T cell:target cell interaction influence a receptor's bioactivity, but also additional factors are in play that are not entirely understood or fully considered yet. It is likely that e.g., CAR flexibility/rigidity and surface stability may have a greater relevance than previously assumed. For instance, Patel and colleagues have shown that the spacer domain can diminish a CAR's functionality by increasing its turnover rate (17). Thus, it is important to take into consideration that Ig domains as they are present in IgG and Siglec spacer domains display a distinct structural folding while the CD8α spacer is derived from a stalk connecting an Ig-like domain with the membrane. Attempts to resolve the structure of the CD8α hinge domain were of limited success so far, indicating the relative flexibility of this region (60). The Siglec-4 or the IgG spacers are missing this flexibility and in this way reduce targetable epitopes to the ones located in membrane proximity.

Another important aspect to be taken into consideration is the tendency of the CD8α stalk region to heterodimerize with CD8β, the subunit that contains raft-localizing determinants (61). As lipid rafts contain an accumulation of accessory molecules decisive for signal transduction and the intracellular CD8β domain has been described to promote association with the two crucial players Lck and LAT (62), it is likely that – in the context of cytotoxic T cells – the CD8α spacer region is capable of attenuating the effector function threshold by fostering interaction with downstream signaling molecules. These effects are absent in IgG- and Siglec-based spacers, so that the overall induction of T cell function is likely primarily guided by the number of triggered CAR molecules (Figure S5).

In support of the *in vitro* data, the Siglec-4 spacer CAR displayed a similar anti-tumor efficacy *in vivo* as the cognate CD8α spacer CAR against TSPAN8 and both therapies were superior to the IgG4-based spacer CAR treatment. Taking into account the length of the spacer regions (CD8α: 45aa; Siglec-4: 280 aa; IgG4: 228aa), we could not observe any obvious correlation with CAR potency and rather identified an intrinsic inferiority of the IgG4 spacer *in vivo*. However, in the context of the TSPAN8 targeting, the modified IgG4 spacer CAR showed a much better relative *in vivo* performance to the CD8α spacer compared to the IgG1 spacer performance in the CD20 study. Indeed, the modified IgG4 spacer (25) has now demonstrated good efficacy in ongoing clinical studies (34) indicating that other factors in CAR design such as the scFv binding domain, transmembrane domain or the drug product formulation may also play a role in *in vivo* function and T cell persistence.

Strikingly, however, while the cytotoxic activity was comparable between the CD8α and Siglec-4 spacer CARs, we observed a reduced secretion of pro-inflammatory cytokines and an attenuated upregulation of activation/exhaustion markers such as 4-1BB, LAG3, and PD1 in the Siglec-4 spacer CAR T cells. Moreover, while the proliferative capacity of Siglec-4 CAR T cells was slightly lower compared to CD8α spacer CAR T cells, the Siglec-4 CAR-treated mice featured a trend toward a higher fraction of T_CM_ phenotype within the CAR T cell cohort which is associated with better overall remission and decreased likelihood of relapse in a clinical context.

It is currently widely established that CAR efficacy correlates closely with the development and severity of CRS in the clinic, an adverse event whose management has proven challenging in the clinical setting. Although tocilizumab and glucocorticoids have been described as effective intervention options, finding the right timing for their application represents a big hurdle (1, 63). In fact, too early intervention may jeopardize the therapeutic efficacy and increase the risk of relapse, while too late intervention bears the risk of CRS-induced multi-organ failure and irreversible neurotoxicities resulting in a patient's death (1, 3, 5, 7, 64–68). Thus, a treatment modality that retains the cytotoxic ability of currently approved CAR T cell therapies but attenuates the levels of secreted cytokines may turn engineered T cells not only into a reliable and effective, but also a safer platform. Moreover, a concomitant increase of the memory phenotype in the CAR T cell cohort of the patient holds promise to further increase the therapeutic efficacy while reducing life-threatening side effects.

Although the phenotype of Siglec-4 CAR modified T cells bodes well for future clinical application, what is the potential toxicity profile of this novel spacer structure? The parent protein Siglec-4, also known as myelin-associated glycoprotein (MAG), has been reported to be exclusively produced by myelinating glial cells such as oligodendrocytes in the central nervous system (CNS; 1% of total protein mass) and Schwann cells in the peripheral nervous system (PNS; 0.1% of total protein mass) (69, 70). Its specific expression on the innermost layer of myelin directly opposite to the axon surface supports its crucial role in the stabilization of axon-myelin interactions, the regulation of myelination, and the inhibition of axon regeneration after injury (71–73). These effects have been first described to be mediated by the N-terminal V-set domain of the receptor, as determined by ligand specificity analyses, site-directed mutagenesis and analogy to the crystal structure of Siglec-1 (74–77). In our evaluation of homology studies, we found the protein sequence to be the best conserved among the Siglecs and within mammalian species. Indeed, the highest sequence homology was identified to lie within the first two N-terminal domains of Siglec-4 (78). Consequently, in order to abrogate these interactions, both N-terminal domains were excluded from our CAR spacer design.

More recent studies, however, suggest that an alternative binding domain exists that interacts with the Nogo receptor 1 and 2 (NgR1, NgR2), but not NgR3 (79–82). Deletion analysis demonstrated that while the first three Ig-like C2-set domains (amino acids 17-325) of Siglec-4 are involved in these interactions, C2-set domains 3-5 (amino acids 234-506) as they are present in our CAR architecture fail to associate with NgR1 or NgR2 (83) indicating that domains 1 and 2 are the major interaction partners. Interestingly, both a soluble and membrane-bound receptor construct comprising the C2-set domains 3-5 of Siglec-4 (amino acids 234-506) are still able to inhibit neurite outgrowth in the CNS, suggesting the existence of an as of yet unidentified ligand partner (83, 84). This observation may indicate the potential risk of unwanted interactions of the Siglec-4 spacer-based CAR T cells with this unknown binding partner. Although the CNS is an immune-privileged organ an intensive infiltration by CAR T cells has been shown to occur as a result of blood-brain-barrier (BBB) damage due to strong CRS. However, as Siglec-4 spacer CAR T cells appear to produce lower levels of cytokines, BBB disruption is expected to be mitigated, in this way minimizing CNS accessibility for CAR T cells.

In support of this hypothesis, despite the high homology between human and rodent Siglec-4 of 95% at the amino acid level over the entire extracellular domain (85, 86), we did not observe any toxicities in the mouse cohort receiving Siglec-4 spacer CAR therapy in our *in vivo* studies. Nevertheless, since - to the best of our knowledge - human-mouse cross-reactivity of Siglec-4 and its interaction partners has not been determined, these data need to be handled with care and further analysis is required to investigate the extent of potential side-effects of Siglec-4 spacer-based CAR T cells.

In summary, this study introduces the new class of Siglec CAR spacers, which structurally resemble IgG class spacers without their FcγR binding features. The Siglec-4 spacer proved to be as efficient as a conventional CD8α spacer in both *in vitro* and *in vivo* CAR function, but exhibited advantageous traits in terms of the T cell phenotype and CAR T cell cytokine release, which make it an interesting candidate CAR structure to translate into future clinical applications.

## Data Availability Statement

All datasets generated for this study are included in the article/Supplementary Material.

## Ethics Statement

The studies involving human participants were reviewed and approved by German Red Cross Dortmund, University Hospital Cologne and German Red Cross Ulm. The patients/participants provided their written informed consent to participate in this study. The animal study was reviewed and approved by Landesamt für Natur, Umwelt, and Verbraucherschutz NRW, Approval numbers 84-02.04.2015.A168 and 84-02.04.2017.A021.

## Author Contributions

RP designed the Siglec based spacers. DS and RP designed the Siglec spacer-based CARs. DS, JH, RP, WA, IJ, and OH designed the study. DS, JH, AS, JB, CB, DG, and WA conducted experiments. DS, JH, and RP wrote the manuscript with input from all authors. NM-T, RP, WA, IJ, and OH supervised the project. All authors discussed the data and reviewed the manuscript.

## Conflict of Interest

DS, JH, RP, JB, NM-T, CB, DG, WA, IJ, and OH are employees of Miltenyi Biotec B.V. & Co. KG. Patent applications are pending to this work. The remaining author declares that the research was conducted in the absence of any commercial or financial relationships that could be construed as a potential conflict of interest.

## References

[1] Lee DW Kochenderfer JN Stetler-Stevenson M Cui YK Delbrook C Feldman SA. T cells expressing CD19 chimeric antigen receptors for acute lymphoblastic leukaemia in children and young adults: a phase 1 dose-escalation trial. Lancet. (2015) 385:517–28. 10.1016/S0140-6736(14)61403-3 25319501 PMC7065359

[2] Porter DL Hwang WT Frey NV Lacey SF Shaw PA Loren AW. Chimeric antigen receptor T cells persist and induce sustained remissions in relapsed refractory chronic lymphocytic leukemia. Sci Transl Med. (2015) 7:303ra139. 10.1126/scitranslmed.aac5415 26333935 PMC5909068

[3] Turtle CJ Hanafi L-A Berger C Gooley TA Cherian S Hudecek M. CD19 CAR-T cells of defined CD4+:CD8+ composition in adult B cell ALL patients. J Clin Investig. (2016) 126:2123–38. 10.1172/JCI85309 27111235 PMC4887159

[4] Neelapu SS Locke FL Bartlett NL Lekakis LJ Miklos DB Jacobson CA. Axicabtagene ciloleucel CAR T-cell therapy in refractory large B-cell lymphoma. N Engl J Med. (2017) 377:2531–44. 10.1056/NEJMoa1707447 29226797 PMC5882485

[5] Schuster SJ Svoboda J Chong EA Nasta SD Mato AR Anak Ö. Chimeric antigen receptor t cells in refractory B-cell lymphomas. N Engl J Med. (2017) 377:2545–54. 10.1056/NEJMoa1708566 29226764 PMC5788566

[6] Maude SL Laetsch TW Buechner J Rives S Boyer M Bittencourt H. Tisagenlecleucel in children and young adults with B-cell lymphoblastic leukemia. N Engl J Med. (2018) 378:439–48. 10.1056/NEJMoa1709866 29385370 PMC5996391

[7] Park JH Rivière I Gonen M Wang X Sénéchal B Curran KJ. Long-term follow-up of CD19 CAR therapy in acute lymphoblastic leukemia. N Engl J Med. (2018) 378:449–59. 10.1056/NEJMoa1709919 29385376 PMC6637939

[8] Gross G Waks T Eshhar Z. Expression of immunoglobulin-T-cell receptor chimeric molecules as functional receptors with antibody-type specificity. Proc Natl Acad Sci USA. (1989) 86:10024–8. 10.1073/pnas.86.24.10024 2513569 PMC298636

[9] Moritz D Groner B. A spacer region between the single chain antibody- and the CD3 zeta-chain domain of chimeric T cell receptor components is required for efficient ligand binding and signaling activity. Gene Therap. (1995) 2:539–46. 8593604

[10] Darcy PK Kershaw MH Trapani JA Smyth MJ. Expression in cytotoxic T lymphocytes of a single-chain anti-carcinoembryonic antigen antibody. Redirected Fas ligand-mediated lysis of colon carcinoma. Euro J Immunol. (1998) 28:1663–72. 10.1002/sici1521-414119980528:051663::aid-immu16633.0.co;2-l 9603473

[11] Eshhar Z Waks T Bendavid A Schindler DG. Functional expression of chimeric receptor genes in human T cells. J Immunol Methods. (2001) 248:67–76. 10.1016/s0022-17590000343-4 11223069

[12] Niederman TMJ Ghogawala Z Carter BS Tompkins HS Russell MM Mulligan RC. Antitumor activity of cytotoxic T lymphocytes engineered to target vascular endothelial growth factor receptors. Proc Natl Acad Sci. (2002) 99:7009–14. 10.1073/pnas.092562399 11997459 PMC124519

[13] Zhang L Lizzio EF Chen T Kozlowski S. Peptide immunization excludes antigen-specific T cells from splenic lymphoid compartments. Euro J Immunol. (2005) 35:776–85. 10.1002/eji.200425479 15714585

[14] Morgenroth A Cartellieri M Schmitz M Günes S Weigle B Bachmann M. Targeting of tumor cells expressing the prostate stem cell antigen. (PSCA) using genetically engineered T-cells. Prostate. (2007) 67:1121–31. 10.1002/pros.20608 17492652

[15] Zhang H Snyder KM Suhoski MM Maus MV Kapoor V June CH. 4-1BB is superior to CD28 costimulation for generating CD8^+^ cytotoxic lymphocytes for adoptive immunotherapy. J Immunol. (2007) 179:4910–8. 10.4049/jimmunol.179.7.4910 17878391 PMC3809056

[16] Barber A Zhang T Megli CJ Wu J Meehan KR Sentman CL. Chimeric NKG2D receptor-expressing T cells as an immunotherapy for multiple myeloma. Exp Hematol. (2008) 36:1318–28. 10.1016/j.exphem.2008.04.010 18599182 PMC2638591

[17] Patel SD Moskalenko M Smith D Maske B Finer MH McArthur JG. Impact of chimeric immune receptor extracellular protein domains on T cell function. Gene Therap. (1999) 6:412–9. 10.1038/sj.gt.3300831 10435091

[18] Guest RD Hawkins RE Kirillova N Cheadle EJ Arnold J O'Neill A. The role of extracellular spacer regions in the optimal design of chimeric immune receptors: evaluation of four different scfvs and antigens. J Immunother. (2005) 28:203–11. 10.1097/01.cji.0000161397.96582.59 15838376

[19] Haso W Lee DW Shah NN Stetler-Stevenson M Yuan CM Pastan IH. Anti-CD22-chimeric antigen receptors targeting B-cell precursor acute lymphoblastic leukemia. Blood. (2013) 121:1165–74. 10.1182/blood-2012-06-438002 23243285 PMC3575759

[20] Hudecek M Lupo-Stanghellini M-T Kosasih PL Sommermeyer D Jensen MC Rader C. Receptor affinity and extracellular domain modifications affect tumor recognition by ROR1-specific chimeric antigen receptor T cells. Clin Cancer Res. (2013) 19:3153–64. 10.1158/1078-0432.CCR-13-0330 23620405 PMC3804130

[21] James SE Greenberg PD Jensen MC Lin Y Wang J Till BG. Antigen sensitivity of CD22-specific chimeric TCR is modulated by target epitope distance from the cell membrane. J Immunol. (2008) 180:7028–38. 10.4049/jimmunol.180.10.7028 18453625 PMC2585549

[22] Krenciute G Krebs S Torres D Wu M-F Liu H Dotti G. Characterization and functional analysis of scFv-based chimeric antigen receptors to redirect T cells to IL13Rα2-positive glioma. Mol Thera. (2016) 24:354–63. 10.1038/mt.2015.199 26514825 PMC4817815

[23] Hombach A Hombach AA Abken H. Adoptive immunotherapy with genetically engineered T cells: modification of the IgG1 Fc 'spacer' domain in the extracellular moiety of chimeric antigen receptors avoids 'off-target' activation and unintended initiation of an innate immune response. Gene Thera. (2010) 17:1206–13. 10.1038/gt.2010.91 20555360

[24] Almåsbak H Walseng E Kristian A Myhre MR Suso EM Munthe LA. Inclusion of an IgG1-Fc spacer abrogates efficacy of CD19 CAR T cells in a xenograft mouse model. Gene Thera. (2015) 22:391–403. 10.1038/gt.2015.4 25652098

[25] Hudecek M Sommermeyer D Kosasih PL Silva-Benedict A Liu L Rader C. The nonsignaling extracellular spacer domain of chimeric antigen receptors is decisive for *in vivo* antitumor activity. Cancer Immunol Res. (2015) 3:125–35. 10.1158/2326-6066.CIR-14-0127 25212991 PMC4692801

[26] Watanabe N Bajgain P Sukumaran S Ansari S Heslop HE Rooney CM. Fine-tuning the CAR spacer improves T-cell potency. Oncoimmunology. (2016) 5:e1253656. 10.1080/2162402X.2016.1253656 28180032 PMC5214260

[27] Jonnalagadda M Mardiros A Urak R Wang X Hoffman LJ Bernanke A. Chimeric antigen receptors with mutated IgG4 Fc spacer avoid fc receptor binding and improve T cell persistence and antitumor efficacy. Mol Thera. (2015) 23:757–68. 10.1038/mt.2014.208 25366031 PMC4395772

[28] Jensen MC Popplewell L Cooper LJ DiGiusto D Kalos M Ostberg JR. Antitransgene rejection responses contribute to attenuated persistence of adoptively transferred CD20/CD19-specific chimeric antigen receptor redirected T cells in humans. Biol Blood Marrow Transplant. (2010) 16:1245–56. 10.1016/j.bbmt.2010.03.014 20304086 PMC3383803

[29] Savoldo B Ramos CA Liu E Mims MP Keating MJ Carrum G. CD28 costimulation improves expansion and persistence of chimeric antigen receptor-modified T cells in lymphoma patients. J Clin Investig. (2011) 121:1822–6. 10.1172/JCI46110 21540550 PMC3083795

[30] Till BG Jensen MC Wang J Qian X Gopal AK Maloney DG. CD20-specific adoptive immunotherapy for lymphoma using a chimeric antigen receptor with both CD28 and 4-1BB domains: pilot clinical trial results. Blood. (2012) 119:3940–50. 10.1182/blood-2011-10-387969 22308288 PMC3350361

[31] Gargett T Yu W Dotti G Yvon ES Christo SN Hayball JD. GD2-specific CAR T cells undergo potent activation and deletion following antigen encounter but can be protected from activation-induced cell death by PD-1 blockade. Mol Therap. (2016) 24:1135–49. 10.1038/mt.2016.63 27019998 PMC4923328

[32] Brown CE Alizadeh D Starr R Weng L Wagner JR Naranjo A. Regression of glioblastoma after chimeric antigen receptor T-cell therapy. N Engl J Med. (2016) 375:2561–9. 10.1056/NEJMoa1610497 28029927 PMC5390684

[33] Wang X Popplewell LL Wagner JR Naranjo A Blanchard MS Mott MR. Phase 1 studies of central memory–derived CD19 CAR T–cell therapy following autologous HSCT in patients with B-cell NHL. Blood. (2016) 127:2980–90. 10.1182/blood-2015-12-686725 27118452 PMC4911862

[34] Abramson JS Palomba ML Gordon LI Lunning MA Wang ML Arnason JE Pivotal safety and efficacy results from transcend NHL 001, a multicenter phase 1 study of lisocabtagene maraleucel (liso-cel) in relapsed/refractory (R/R) large B cell lymphomas. Blood. (2019) 134(Suppl.1):241 10.1182/blood-2019-127508

[35] Crocker PR Varki A. Siglecs, sialic acids and innate immunity. Trends Immunol. (2001) 22:337–42. 10.1016/s1471-4906(01)01930-5 11377294

[36] Crocker PR Paulson JC Varki A. Siglecs and their roles in the immune system. Nat Rev Immunol. (2007) 7:255–66. 10.1038/nri2056 17380156

[37] Jensen M Tan G Forman S Wu AM Raubitschek A. CD20 is a molecular target for scFvFc:zeta receptor redirected T cells: implications for cellular immunotherapy of CD20+ malignancy. Biol Blood Marrow Transplant. (1998) 4:75–83. 10.1053/bbmt.1998.v4.pm9763110 9763110

[38] Apel M Brüning M Granzin M Essl M Stuth J Blaschke J Integrated clinical scale manufacturing system for cellular products derived by magnetic cell separation, centrifugation and cell culture. Chem Ingen Tech. (2013) 85:103–10. 10.1002/cite.201200175

[39] Lock D Mockel-Tenbrinck N Drechsel K Barth C Mauer D Schaser T. Automated manufacturing of potent CD20-directed chimeric antigen receptor t cells for clinical use. Hum Gene Ther. (2017) 28:914–25. 10.1089/hum.2017.111 28847167

[40] Al Rawashdeh W Zuo S Melle A Appold L Koletnik S Tsvetkova Y. Noninvasive assessment of elimination and retention using CT-FMT and kinetic whole-body modeling. Theranostics. (2017) 7:1499–510. 10.7150/thno.17263 28529633 PMC5436509

[41] Till BG Jensen MC Wang J Chen EY Wood BL Greisman HA. Adoptive immunotherapy for indolent non-hodgkin lymphoma and mantle cell lymphoma using genetically modified autologous CD20-specific T cells. Blood. (2008) 112:2261–71. 10.1182/blood-2007-12-128843 18509084 PMC2532803

[42] Polyak MJ Deans JP. Alanine-170 and proline-172 are critical determinants for extracellular CD20 epitopes; heterogeneity in the fine specificity of CD20 monoclonal antibodies is defined by additional requirements imposed by both amino acid sequence and quaternary structure. Blood. (2002) 99:3256–62. 10.1182/blood.v99.9.3256 11964291

[43] Macauley MS Crocker PR Paulson JC. Siglec-mediated regulation of immune cell function in disease. Nat Rev Immunol. (2014) 14:653–66. 10.1038/nri3737 25234143 PMC4191907

[44] Bornhöfft KF Goldammer T Rebl A Galuska SP. Siglecs: a journey through the evolution of sialic acid-binding immunoglobulin-type lectins. Dev Comparat Immunol. (2018) 86:219–31. 10.1016/j.dci.2018.05.008 29751010

[45] Srivastava S Riddell SR. Engineering CAR-T cells: design concepts. Trends Immunol. (2015) 36:494–502. 10.1016/j.it.2015.06.004 26169254 PMC4746114

[46] Riddell SR Elliott M Lewinsohn DA Gilbert MJ Wilson L Manley SA. T-cell mediated rejection of gene-modified HIV-specific cytotoxic T lymphocytes in HIV-infected patients. Nat Med. (1996) 2:216–23. 10.1038/nm0296-216 8574968

[47] Berger C Flowers ME Warren EH Riddell SR. Analysis of transgene-specific immune responses that limit the *in vivo* persistence of adoptively transferred HSV-TK-modified donor T cells after allogeneic hematopoietic cell transplantation. Blood. (2006) 107:2294–302. 10.1182/blood-2005-08-3503 16282341 PMC1895724

[48] Hernández-Caselles T Martínez-Esparza M Pérez-Oliva AB Quintanilla-Cecconi AM García-Alonso A Alvarez-López DMR. A study of CD33. (SIGLEC-3) antigen expression and function on activated human T and NK cells: two isoforms of CD33 are generated by alternative splicing. J Leukocyte Biol. (2006) 79:46–58. 10.1189/jlb.0205096 16380601

[49] Laszlo GS Harrington KH Gudgeon CJ Beddoe ME Fitzgibbon MP Ries RE. Expression and functional characterization of CD33 transcript variants in human acute myeloid leukemia. Oncotarget. (2016) 7:43281–94. 10.18632/oncotarget.9674 27248327 PMC5190023

[50] Pérez-Oliva AB Martínez-Esparza M Vicente-Fernández JJ Corral-San Miguel R García-Peñarrubia P Hernández-Caselles T. Epitope mapping, expression and post-translational modifications of two isoforms of CD33 (CD33M and CD33m) on lymphoid and myeloid human cells. Glycobiology. (2011) 21:757–70. 10.1093/glycob/cwq220 21278227

[51] Künkele A Johnson AJ Rolczynski LS Chang CA Hoglund V Kelly-Spratt KS. Functional tuning of CARs reveals signaling threshold above which CD8+ CTL antitumor potency is attenuated due to cell Fas-FasL-dependent AICD. Cancer Immunol Res. (2015) 3:368–79. 10.1158/2326-6066.CIR-14-0200 25576337

[52] Valitutti S Müller S Dessing M Lanzavecchia A. Signal extinction and T cell repolarization in T helper cell-antigen-presenting cell conjugates. Euro J Immunol. (1996) 26:2012–6. 10.1002/eji.1830260907 8814239

[53] Itoh Y Germain RN. Single cell analysis reveals regulated hierarchical T cell antigen receptor signaling thresholds and intraclonal heterogeneity for individual cytokine responses of CD4+ T cells. J Exp Med. (1997) 186:757–66. 10.1084/jem.186.5.757 9271591 PMC2199012

[54] Hemmer B Vergelli M Gran B Ling N Conlon P Pinilla C. Predictable TCR antigen recognition based on peptide scans leads to the identification of agonist ligands with no sequence homology. J Immunol. (1998) 160:3631–6 9558061

[55] Auphan-Anezin N Verdeil G Schmitt-Verhulst A-M. Distinct thresholds for CD8 T cell activation lead to functional heterogeneity: CD8 T cell priming can occur independently of cell division. J Immunol. (2003) 170:2442–8. 10.4049/jimmunol.170.5.2442 12594268

[56] Faroudi M Zaru R Paulet P Müller S Valitutti S. Cutting edge: T lymphocyte activation by repeated immunological synapse formation and intermittent signaling. J Immunol. (2003) 171:1128–32. 10.4049/jimmunol.171.3.1128 12874197

[57] Liu Z Gerner MY Van Panhuys N Levine AG Rudensky AY Germain RN. Immune homeostasis enforced by co-localized effector and regulatory T cells. Nature. (2015) 528:225–30. 10.1038/nature16169 26605524 PMC4702500

[58] Caruso HG Hurton LV Najjar A Rushworth D Ang S Olivares S. Tuning sensitivity of CAR to EGFR density limits recognition of normal tissue while maintaining potent antitumor activity. Cancer Res. (2015) 75:3505–18. 10.1158/0008-5472.can-15-0139 26330164 PMC4624228

[59] Ghorashian S Kramer AM Onuoha S Wright G Bartram J Richardson R. Enhanced CAR T cell expansion and prolonged persistence in pediatric patients with ALL treated with a low-affinity CD19 CAR. Nat Med. (2019) 25:1408–14. 10.1038/s41591-019-0549-5 31477906

[60] Leahy DJ Axel R Hendrickson WA. Crystal structure of a soluble form of the human T cell coreceptor CD8 at 2.6 a resolution. Cell. (1992) 68:1145–62. 10.1016/0092-8674(92)90085-q 1547508

[61] Pang S Zhang L Wang H Yi Z Li L Gao L. CD8(+) T cells specific for beta cells encounter their cognate antigens in the islets of NOD mice. Euro J Immunol. (2009) 39:2716–24. 10.1002/eji.200939408 19658094

[62] Bosselut R Kubo S Guinter T Kopacz JL Altman JD Feigenbaum L. Role of CD8β domains in CD8 coreceptor function: importance for MHC I binding, signaling, and positive selection of CD8+ T cells in the thymus. Immunity. (2000) 12:409–18. 10.1016/S1074-7613(00)80193-4 10795739

[63] Brudno JN Kochenderfer JN. Toxicities of chimeric antigen receptor T cells: recognition and management. Blood. (2016) 127:3321–30. 10.1182/blood-2016-04-703751 27207799 PMC4929924

[64] Brentjens RJ Davila ML Riviere I Park J Wang X Cowell LG. CD19-targeted T cells rapidly induce molecular remissions in adults with chemotherapy-refractory acute lymphoblastic leukemia. Sci Transl Med. (2013) 5:177ra138. 10.1126/scitranslmed.3005930 23515080 PMC3742551

[65] Grupp SA Kalos M Barrett D Aplenc R Porter DL Rheingold SR. Chimeric antigen receptor-modified T cells for acute lymphoid leukemia. N Engl J Med. (2013) 368:1509–18. 10.1056/NEJMoa1215134 23527958 PMC4058440

[66] Maude SL Barrett D Teachey DT Grupp SA. Managing cytokine release syndrome associated with novel T cell-engaging therapies. Cancer J. (2014) 20:119–22. 10.1097/PPO.0000000000000035 24667956 PMC4119809

[67] Kochenderfer JN Dudley ME Kassim SH Somerville RP Carpenter RO Stetler-Stevenson M. Chemotherapy-refractory diffuse large B-cell lymphoma and indolent B-cell malignancies can be effectively treated with autologous T cells expressing an anti-CD19 chimeric antigen receptor. J Clin Oncol. (2015) 33:540–9. 10.1200/jco.2014.56.2025 25154820 PMC4322257

[68] Hay KA Hanafi L-A Li D Gust J Liles WC Wurfel MM. Kinetics and biomarkers of severe cytokine release syndrome after CD19 chimeric antigen receptor-modified T-cell therapy. Blood. (2017) 130:2295–306. 10.1182/blood-2017-06-793141 28924019 PMC5701525

[69] Bartsch U Kirchhoff F Schachner M. Immunohistological localization of the adhesion molecules L1, N-CAM, and MAG in the developing and adult optic nerve of mice. J Comparat Neurol. (1989) 284:451–62. 10.1002/cne.902840310 2474006

[70] Trapp BD Andrews SB Cootauco C Quarles R. The myelin-associated glycoprotein is enriched in multivesicular bodies and periaxonal membranes of actively myelinating oligodendrocytes. J Cell Biol. (1989) 109:2417–26. 10.1083/jcb.109.5.2417 2478568 PMC2115868

[71] McKerracher L David S Jackson DL Kottis V Dunn RJ Braun PE. Identification of myelin-associated glycoprotein as a major myelin-derived inhibitor of neurite growth. Neuron. (1994) 13:805–11. 10.1016/0896-6273(94)90247-x 7524558

[72] Mukhopadhyay G Doherty P Walsh FS Crocker PR Filbin MT. A novel role for myelin-associated glycoprotein as an inhibitor of axonal regeneration. Neuron. (1994) 13:757–67. 10.1016/0896-62739490042-6 7522484

[73] Schachner M Bartsch U. Multiple functions of the myelin-associated glycoprotein MAG (siglec-4a) in formation and maintenance of myelin. Glia. (2000) 29:154–65. 10.1002/sici1098-11362000011529:2154::aid-glia93.0.co;2-3 10625334

[74] Collins BE Ito H Sawada N Ishida H Kiso M Schnaar RL. Enhanced binding of the neural siglecs, myelin-associated glycoprotein and Schwann cell myelin protein, to Chol-1 (alpha-series) gangliosides and novel sulfated Chol-1 analogs. J Biol Chem. (1999) 274:37637–43. 10.1074/jbc.274.53.37637 10608819

[75] Tang S Shen YJ DeBellard ME Mukhopadhyay G Salzer JL Crocker PR. Myelin-associated glycoprotein interacts with neurons via a sialic acid binding site at ARG118 and a distinct neurite inhibition site. J Cell Biol. (1997) 138:1355–66. 10.1083/jcb.138.6.1355 9298990 PMC2132563

[76] May AP Robinson RC Vinson M Crocker PR Jones EY. Crystal structure of the N-terminal domain of sialoadhesin in complex with 3' sialyllactose at 1.85 A resolution. Mol Cell. (1998) 1:719–28. 10.1016/s1097-2765(00)80071-4 9660955

[77] Zaccai NR Maenaka K Maenaka T Crocker PR Brossmer R Kelm S. Structure-guided design of sialic acid-based siglec inhibitors and crystallographic analysis in complex with sialoadhesin. Structure. (2003) 11:557–67. 10.1016/s0969-2126(03)00073-x 12737821

[78] Lehmann F Gäthje H Kelm S Dietz F. Evolution of sialic acid–binding proteins: molecular cloning and expression of fish siglec-4. Glycobiology. (2004) 14:959–68. 10.1093/glycob/cwh120 15229193

[79] Domeniconi M Cao Z Spencer T Sivasankaran R Wang K Nikulina E. Myelin-associated glycoprotein interacts with the Nogo66 receptor to inhibit neurite outgrowth. Neuron. (2002) 35:283–90. 10.1016/s0896-6273(02)00770-5 12160746

[80] Liu BP Fournier A GrandPré T Strittmatter SM. Myelin-associated glycoprotein as a functional ligand for the Nogo-66 receptor. Science. (2002) 297:1190–3. 10.1126/science.1073031 12089450

[81] Venkatesh K Chivatakarn O Lee H Joshi PS Kantor DB Newman BA. The Nogo-66 receptor homolog NgR2 is a sialic acid-dependent receptor selective for myelin-associated glycoprotein. J Neurosci. (2005) 25:808–22. 10.1523/jneurosci.4464-04.2005 15673660 PMC6725623

[82] Atwal JK Pinkston-Gosse J Syken J Stawicki S Wu Y Shatz C. PirB is a functional receptor for myelin inhibitors of axonal regeneration. Science. (2008) 322:967. 10.1126/science.1161151 18988857

[83] Robak LA Venkatesh K Lee H Raiker SJ Duan Y Lee-Osbourne J. Molecular basis of the interactions of the Nogo-66 receptor and its homolog NgR2 with myelin-associated glycoprotein: development of NgROMNI-Fc, a novel antagonist of CNS myelin inhibition. J Neurosci. (2009) 29:5768–83. 10.1523/JNEUROSCI.4935-08.2009 19420245 PMC2779053

[84] Cao Z Qiu J Domeniconi M Hou J Bryson JB Mellado W. The inhibition site on myelin-associated glycoprotein is within Ig-domain 5 and is distinct from the sialic acid binding site. J Neurosci. (2007) 27:9146–54. 10.1523/jneurosci.2404-07.2007 17715351 PMC6672207

[85] Arquint M Roder J Chia LS Down J Wilkinson D Bayley H. Molecular cloning and primary structure of myelin-associated glycoprotein. Proc Natl Acad Sci USA. (1987) 84:600–4. 10.1073/pnas.84.2.600 2432614 PMC304258

[86] Lai C Brow MA Nave KA Noronha AB Quarles RH Bloom FE. Two forms of 1B236/myelin-associated glycoprotein, a cell adhesion molecule for postnatal neural development, are produced by alternative splicing. Proc Natl Acad Sci USA. (1987) 84:4337–41. 10.1073/pnas.84.12.4337 2438699 PMC305080

