# Supplementary material for: A Novel Siglec-4 Derived Spacer Improves the Functionality of CAR T Cells Against Membrane-Proximal Epitopes
Source: Front Immunol. 2020 Aug 7;11:1704. doi: 10.3389/fimmu.2020.01704 (PMC7426717; doi:10.3389/fimmu.2020.01704)
Supplement: Supplementary file 1 [file Data_Sheet_1.PDF]

Supplements

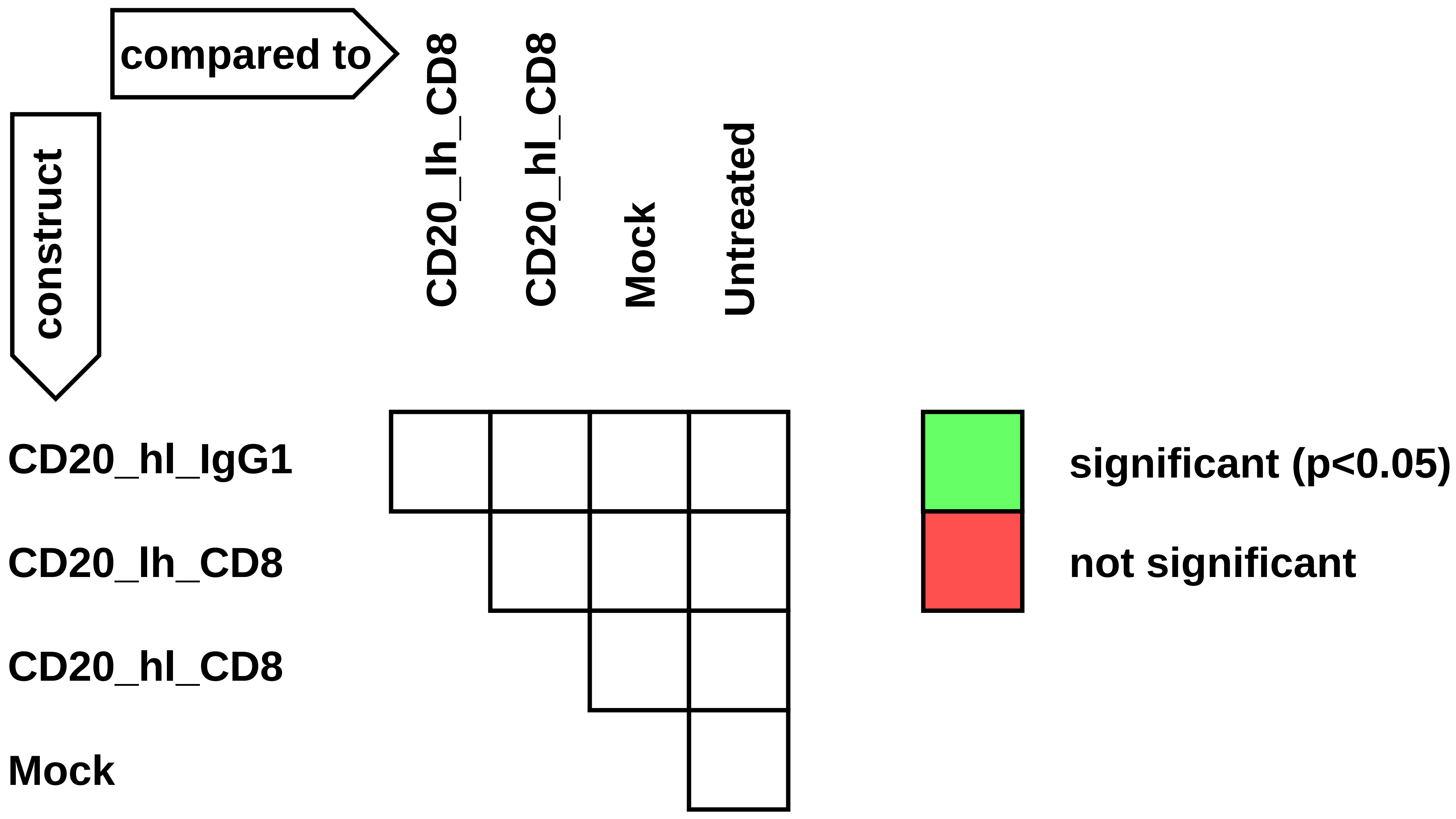

Figure S1: Organization of the pairwise significant matrix for group comparison of *in vivo* performance of CD20_hl_IgG1, CD20_lh_CD8, CD20_hl_CD8, Untreated and Mock. PSM $p<0.05$ (green) [one-way ANOVA, multiple comparisons].

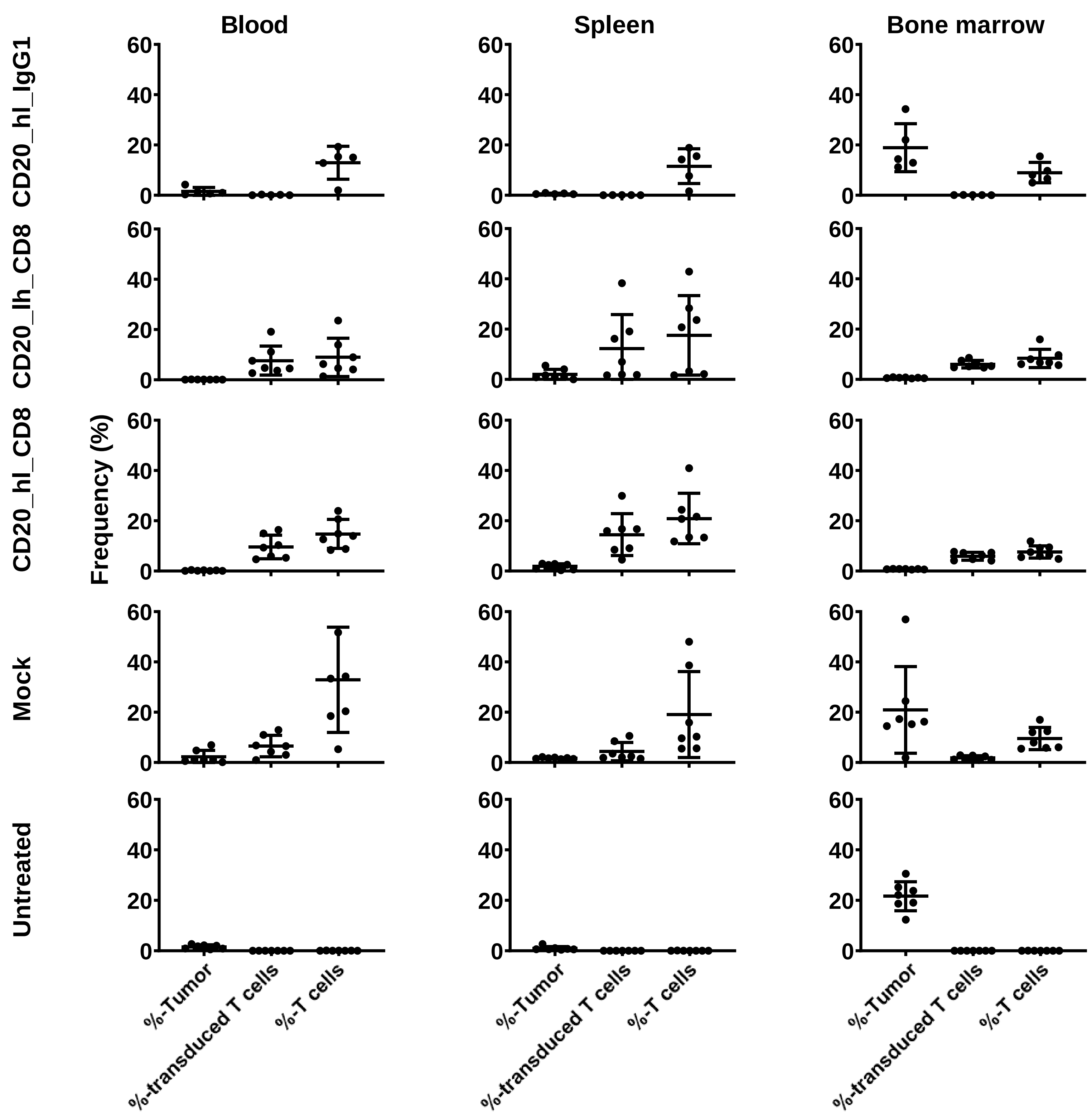

Figure S2: Flow cytometry analysis of tumor and CD20 CAR T cells persistence in blood, spleen and bone marrow. *Ex vivo* analyses were performed at the end point of the study. Percentage of transduced T cells in the Mock group was calculated based on GFP expression. n = 5 – 6.

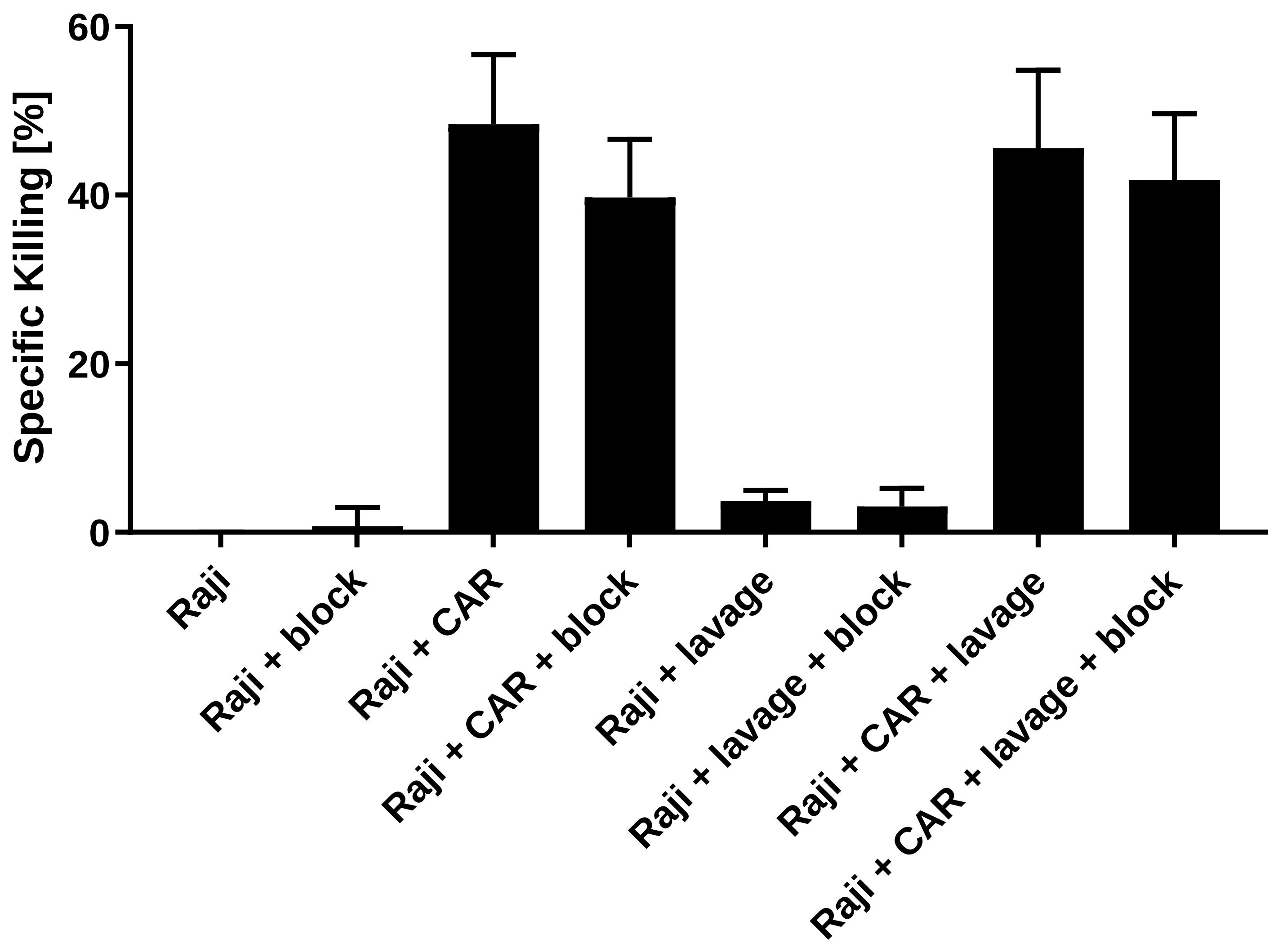

Figure S3: Macrophages do not inhibit the function of CAR T cells containing an IgG1 spacer with mutated Fc-binding sites. CD20_hl_IgG1 CAR T cells were mixed in a 1:1:1 ratio with Raji target cells and NSG macrophages, derived from a peritoneal lavage. Macrophages, tumor and CAR T cells were co-incubated for 24 hours in absence or presence of murine FcR-blocking reagent (block). n = 3.

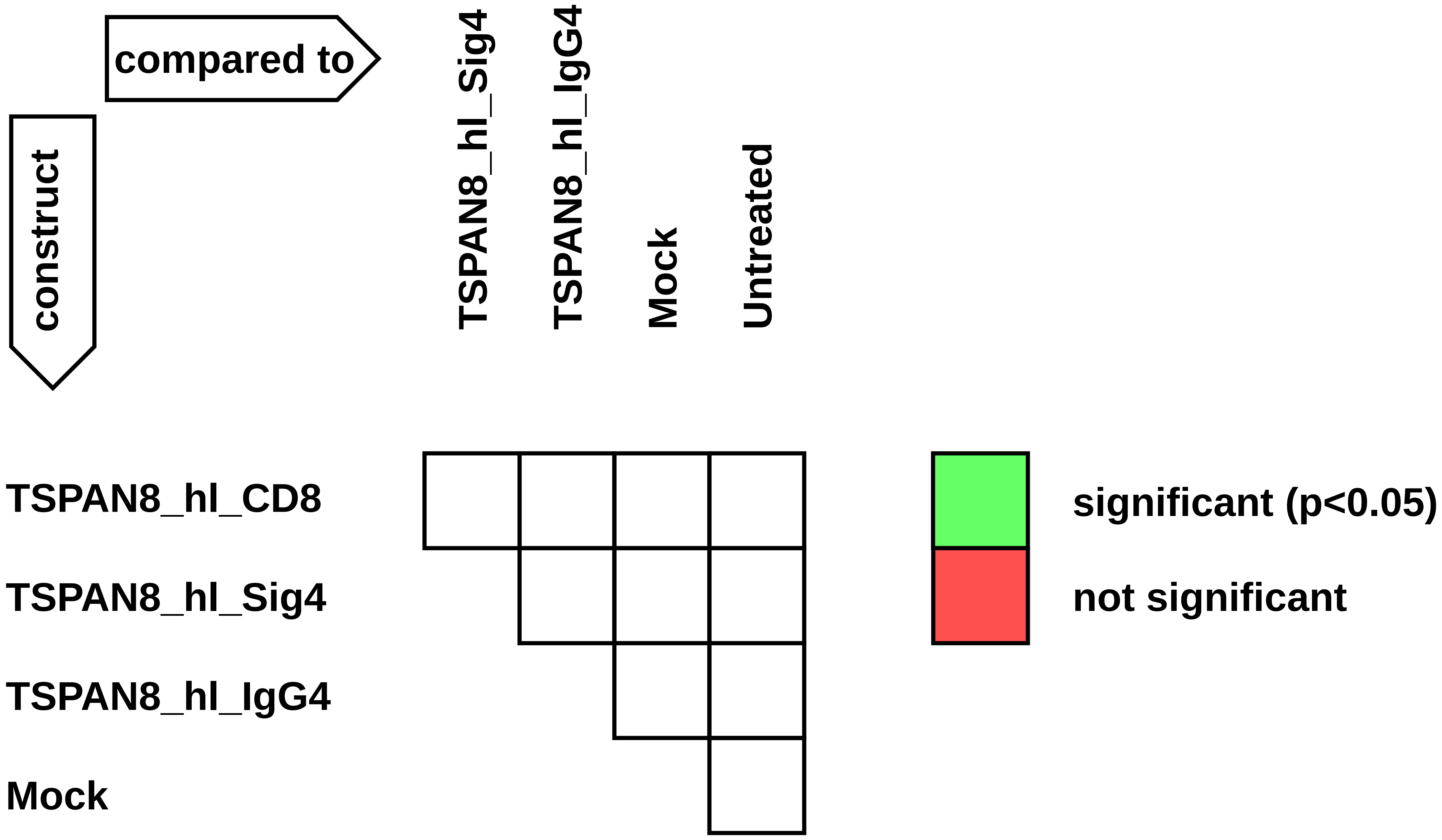

Figure S4: Organization of the pairwise significant matrix for group comparison of *in vivo* performance of TSPAN8_hl_CD8, TSPAN8_hl_Sig4, TSPAN8_hl_IgG4, Untreated and Mock. PSM p<0.05 (green) [one-way ANOVA, multiple comparisons].

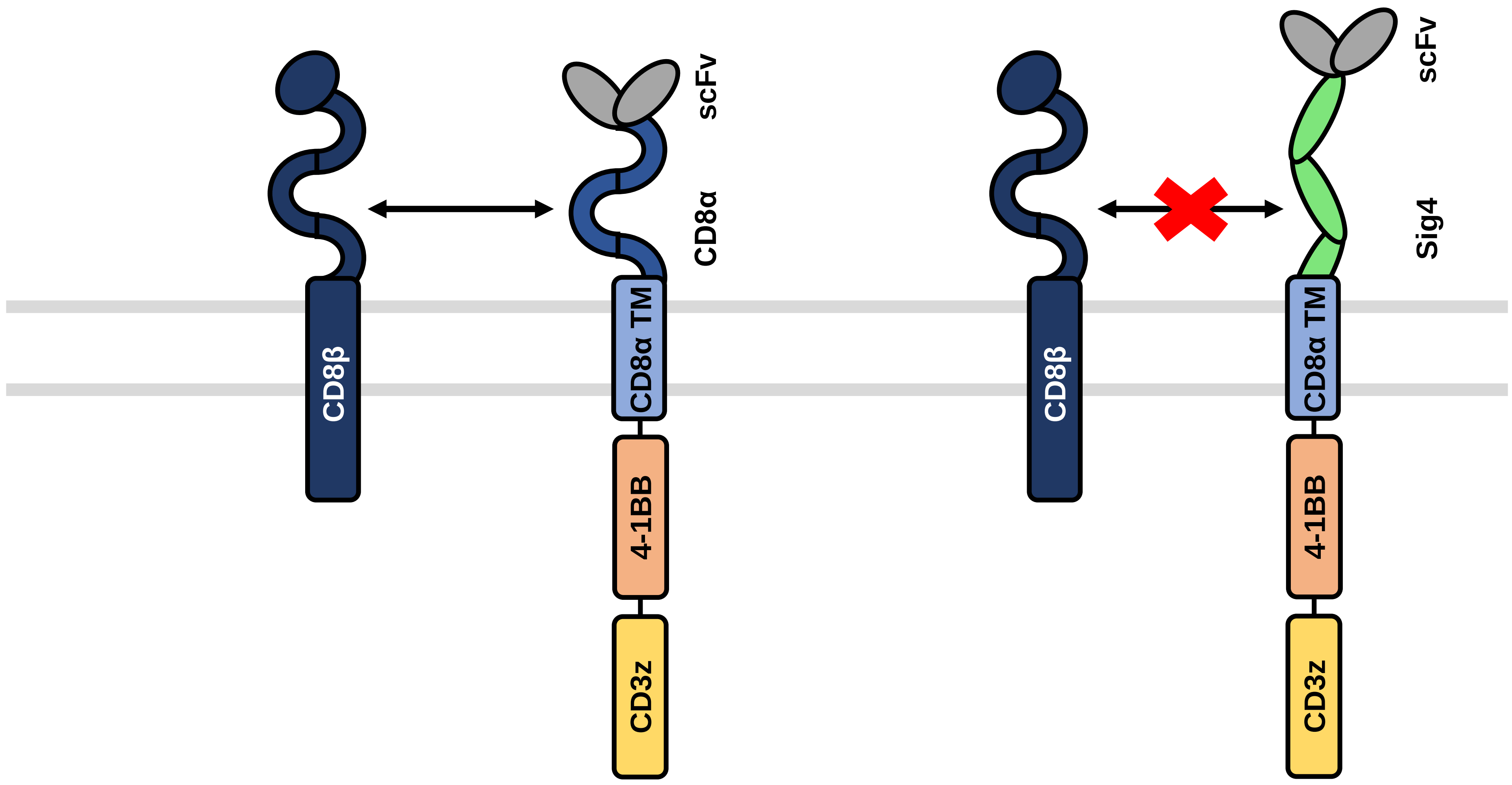

Figure S5: Schematic model of spacer dimerization with natural CD8β leading to increased signaling.
